# Defining the scope for altering rice leaf anatomy to improve photosynthesis: a modelling approach

**DOI:** 10.1111/nph.18564

**Published:** 2022-11-25

**Authors:** Yi Xiao, Jen Sloan, Chris Hepworth, Marc Fradera‐Soler, Andrew Mathers, Rachel Thorley, Alice Baillie, Hannah Jones, Tiangen Chang, Xingyuan Chen, Nazmin Yaapar, Colin P. Osborne, Craig Sturrock, Sacha J. Mooney, Andrew J. Fleming, Xin‐Guang Zhu

**Affiliations:** ^1^ Center of Excellence for Molecular Plant Science, Institute of Plant Physiology and Ecology CAS Shanghai 200032 China; ^2^ Plants, Photosynthesis and Soil, Biosciences University of Sheffield Western Bank Sheffield S10 2TN UK; ^3^ Division of Agriculture and Environmental Sciences, School of Biosciences University of Nottingham Sutton Bonington Campus, Loughborough Leicestershire LE12 5RD UK; ^4^ Pacific Northwest National Laboratory Richland WA 99354 USA; ^5^ Department of Crop Science, Faculty of Agriculture Universiti Putra Malaysia 43400 Serdang Malaysia

**Keywords:** anatomy, leaf, mesophyll, photosynthesis, rice, systems biology

## Abstract

- Leaf structure plays an important role in photosynthesis. However, the causal relationship and the quantitative importance of any single structural parameter to the overall photosynthetic performance of a leaf remains open to debate. In this paper, we report on a mechanistic model, *eLeaf,* which successfully captures rice leaf photosynthetic performance under varying environmental conditions of light and CO_2_.
- We developed a 3D reaction‐diffusion model for leaf photosynthesis parameterised using a range of imaging data and biochemical measurements from plants grown under ambient and elevated CO_2_ and then interrogated the model to quantify the importance of these elements.
- The model successfully captured leaf‐level photosynthetic performance in rice. Photosynthetic metabolism underpinned the majority of the increased carbon assimilation rate observed under elevated CO_2_ levels, with a range of structural elements making positive and negative contributions. Mesophyll porosity could be varied without any major outcome on photosynthetic performance, providing a theoretical underpinning for experimental data.
- *eLeaf* allows quantitative analysis of the influence of morphological and biochemical properties on leaf photosynthesis. The analysis highlights a degree of leaf structural plasticity with respect to photosynthesis of significance in the context of attempts to improve crop photosynthesis.

## Introduction

Photosynthesis occurs primarily in highly organised, multicellular structures, the leaves. Although the process of light harvesting to generate the chemical energy required for subsequent incorporation of gaseous CO_2_ into triose phosphates via the Calvin–Benson cycle is a photobiochemical/metabolic process, it occurs within organelles (chloroplasts), which are localised in cells which are themselves embedded at some distance from the initial incidence of the CO_2_ and light required for photosynthesis. It is thus self‐evident that the physical structures surrounding the chloroplasts within a leaf must, to some extent, limit the process. The identity of these structural elements and their relative importance have been the subject of extensive investigation, leading to a number of important observations and conclusions on leaf structure/function in relation to photosynthesis, and how it responds to altered environmental conditions (Terashima *et al*., 2011; Lundgren & Fleming, 2020).

In addition to simple observations of histology and reasoned interpretations, two main approaches to unravelling this complex problem have been taken. First, investigators have performed large‐scale measurements on multiple leaf structural features in a wide range of related or unrelated species and then performed various correlation analyses to identify potential links between traits and photosynthetic performance. Consequently, huge efforts have been devoted to measuring anatomical features and their proxies and correlating them to photosynthesis (Wright *et al*., 2004; Terashima *et al*., 2011; Giuliani *et al*., 2013; John *et al*., 2017). This has led to the identification of a number of structural parameters relevant to photosynthetic performance, including, for example, exposed mesophyll cell (MC) surface area (*S*_mes_) and the packing of plastids along the plasma membrane. Such correlative approaches have served eco‐physiological studies well but can only provide limited mechanistic evidence on how the identified trait is linked to the output. Consequently, the causal relationship and the quantitative importance of any single structural parameter to the overall photosynthetic performance of a leaf remains open to discussion. The situation is made even more complicated by the fact that the highly diverse combinations of different anatomical and biochemical features within a leaf are liable to interact to determine the final leaf photosynthetic rate. For example, leaf structural features influence both CO_2_ diffusion and light environments inside a leaf, and the distribution of enzymes across a leaf can greatly influence the CO_2_ microclimate, all of which will influence overall photosynthetic performance. Testing the significance of any single factor via, for example, transgenic approaches can lead to many simultaneous structural and biochemical changes, complicating the interpretation of the outcome on photosynthesis.

To tackle these difficulties, some researchers have taken a more mechanistic, bottom‐up approach in which the cellular (and subcellular) structure of a leaf is used as a basis for computational modelling, with quantitative estimates of structure and biochemistry used to investigate whether the integrated activity of multiple cells in a virtual leaf can be used to simulate leaf photosynthetic function (Zhu *et al*., 2013; Ho *et al*., 2016; Xiao *et al*., 2016; Earles *et al*., 2019). Such models inevitably involve a number of assumptions about leaf structure and metabolic properties. However, if the model successfully captures measured reality to a degree of accuracy, it provides evidence that the proposed mechanisms built into the model are to some extent correct. In addition, once validated, these models can allow quantitative dissection of the process under study, assigning values (and thus relative importance) to the different elements comprising the model. Finally, such models are open to the exploration of parameter space in a way which is experimentally very difficult and time‐consuming, thus allowing a more rapid evaluation of hypotheses and setting the scene for targeted experimentation to test interesting or surprising ideas arising from the model.

Developing such models has been a focus of efforts for many years. For example, a complete dynamic systems model of photosynthesis was developed by Zhu *et al*. (2013) although this model did not incorporate any element of 3D leaf structure. 3D leaf models have been developed later for two dicot species, Arabidopsis and tomato (Ho *et al*., 2016; Xiao & Zhu, 2017). In the tomato model, leaf structure was represented as a geometrical representation based on synchrotron radiation X‐ray laminography, with the influence of CO_2_ diffusion on the carbon fixation process simulated via a reaction‐diffusion process, which successfully enabled realistic evaluation of leaf photosynthetic CO_2_ uptake under different CO_2_ and light levels (Ho *et al*., 2016). However, since the structure of the virtual leaf was fixed, this model only allowed change in chloroplast distribution but did not allow the dissection of the relative contribution of different anatomical features to leaf photosynthetic rate. Xiao & Zhu (2017) later developed a reaction‐diffusion model with the leaf structure represented as combinations of mathematical objects. This enabled the identification of anatomical and biochemical factors contributing to mesophyll conductance. However, it was still time‐consuming to build and solve such a model with different geometries, which not only limited its application for a particular leaf but also limited a direct quantification of the contribution of the various leaf structural factors incorporated. Recently, Retta *et al*. (2020) developed a powerful virtual 2D leaf generator to explore the role of leaf structures during growth. However, in this model, the chloroplast structure and vacuole were not incorporated, and the light gradient was assumed to be uniform. Thus, although great strides have been made in the development of computational models which capture elements of photosynthesis at the whole leaf level, a more flexible model, able to allow variation of different elements of leaf structure, as well as allowing simulation of light propagation and CO_2_ diffusion in 3D, would be of benefit to the field. In particular, the ability to interrogate models to explore parameter space as leaf structure varies in response to environmental triggers would open the door to implementing an approach to mechanistically and quantitatively relate leaf structure to function.

**Fig. 1:**
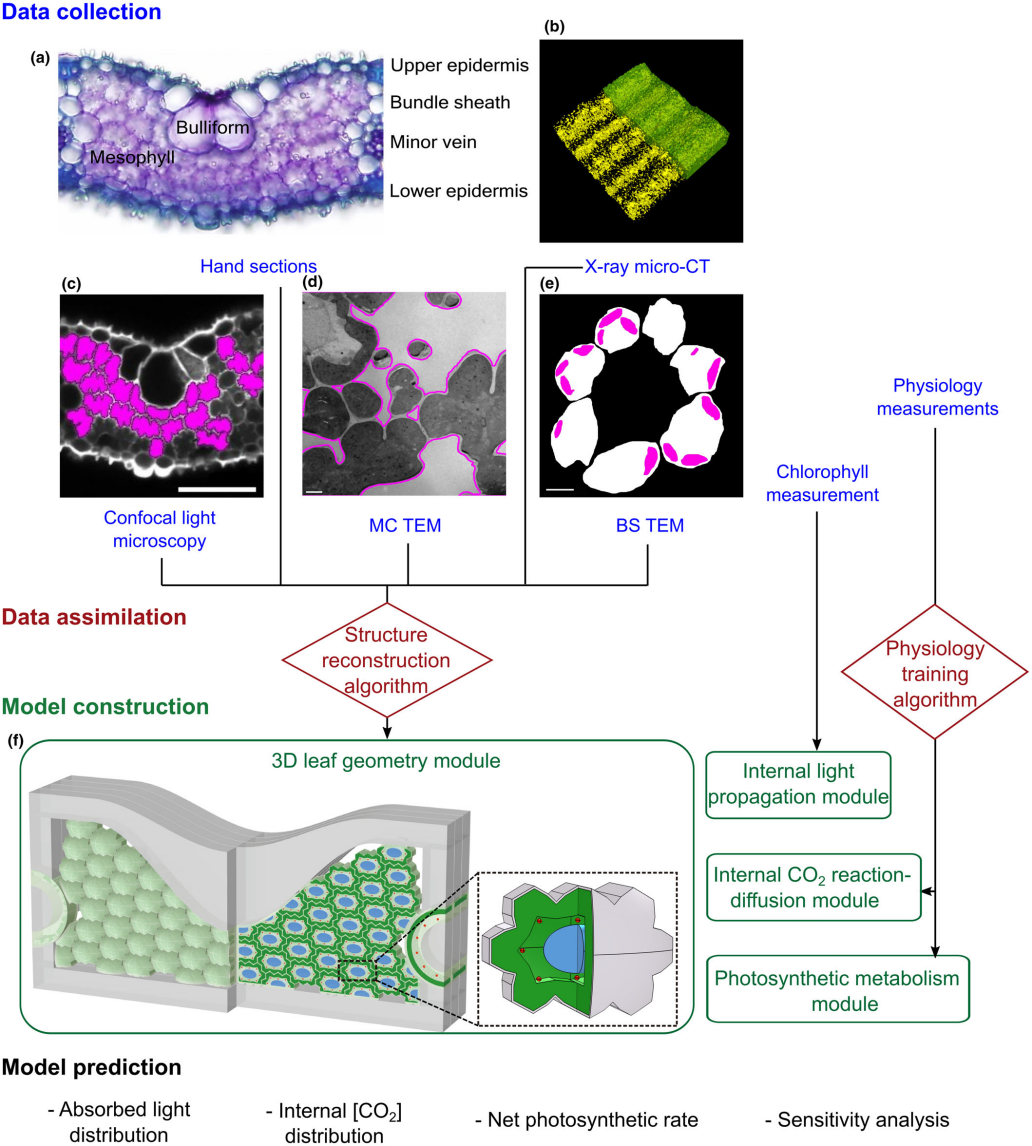
Creation of the *eLeaf* model. (a) Section of a rice leaf showing lobed mesophyll cells, bulliform cells and vasculature surrounded by bundle sheath cells. Data are collected by microCT (b), confocal light microscopy (c) and transmission electron microscopy of thin sections (d, e) to parameterise a modelled leaf (f). This is an abstraction of rice leaf histology in which the macrodimensions of leaf thickness and vein spacing observed in (a) are used to define a skeleton, which is then filled with virtual mesophyll cells (MCs) whose size and shape are informed by measurements in (a, c), as are size parameters of the bundle sheath (BS). At the level of the individual MC (inset f), measurements from (c–e) inform MC length, width and depth, as well as the proportion of the cell occupied by plastids for both MC and BS, and the thickness of the cell wall. The packing of the virtual cells in (f) is dictated to accord with the exposed MC surface area and mesophyll porosity measured in (b). Each virtual cell performs a modelled photosynthetic metabolism based on predicted light propagation and CO_2_ based on a reaction‐diffusion module, parameterised by measurement of biochemical and physiological performance in rice (IR64) leaf 5 at maturity. The model allows the prediction of absorbed light distribution, internal CO_2_ distribution and net photosynthetic rate.

Rice is a major staple crop for a large fraction of the world population. The leaves have a distinctive monocot structure (Fig. 1a) in which similarly shaped, highly lobed MCs are distributed across the adaxial–abaxial axis of the leaf, separating veins which are bounded by bundle sheath (BS) cells at the interface with the mesophyll. The adaxial mesophyll between vascular bundles is distinguished by relatively large bulliform cells. Previous correlation‐based approaches successfully identified various rice leaf structure parameters linked to photosynthetic performance (Giuliani *et al*., 2013); however, to date, no mechanistic reaction‐diffusion‐based model has been developed for rice (or indeed any monocot grass). The development of a mechanistic model would allow *in silico* exploration of the relative contribution of anatomical and metabolic parameters to photosynthetic performance in rice, opening the door to identifying potential modifications for improved photosynthetic efficiency, a major goal of translational research in this area (Evans, 2013; Long *et al*., 2015; Ort *et al*., 2015).

In this paper, we report on the creation of a mechanistic model, *eLeaf,* which successfully captures many elements of rice leaf photosynthetic performance. We use the model to explore how the performance of the leaf changes when plants are grown in elevated CO_2_, an environmental factor of future significance for crop growth which causes both major and more subtle changes in leaf structure (Sanz‐Saez *et al*., 2017; Ainsworth *et al*., 2020). Our modelling allows a quantitative assessment of the impact of leaf structural elements on photosynthesis in rice, and we identify structural components, which can be significantly altered without any great detriment to leaf photosynthetic performance. These data are discussed in the context of the inherent plasticity of leaf structure/function and its potential significance in the context of attempts to improve rice photosynthesis.

## Materials and Methods

### Modelling

The *eLeaf* model comprises four modules, which integrate the measured anatomical and physiological data. These are as follows: (1) a module allowing automatic 3D reconstruction of leaf anatomy based on experimental data; (2) a ‘light’ module consisting of a ray‐tracing algorithm simulating the heterogeneous internal light environment within the leaf; (3) a ‘CO_2_’ module simulating the reaction‐diffusion process of CO_2_ from the subcellular cavities to the chloroplasts of each MC; and (4) a ‘metabolism’ module describing photosynthetic metabolism with the Farquhar–von Caemmerer–Berry (FvCB) model. A description of the *eLeaf* model is provided in Methods S1, as is a user manual for the *eLeaf* code, with abbreviations listed in Table S1.

3D geometries for aCO_2_ and eCO_2_ models were constructed from experimental data (Table S2) using an in‐house package for automatic 3D reconstruction (Video S1). 3D geometries for intermediate models were also constructed where a single group of structural parameters obtained under eCO_2_ were substituted in the aCO_2_ model. With the reconstructed leaf anatomy, the light module explicitly simulated the light reflection, refraction and absorption inside the leaf. Thus, the light absorption of the chloroplast volume is predicted and used to calculate the potential electron transport rate *J* in the ‘metabolism’ module of each cell. The CO_2_ module consisted of the gaseous phase diffusion of CO_2_ and liquid phase diffusion of CO_2_, coupled with diffusion of HCO_3_^−^ through (de)hydration in cells (see Methods S1 for details). This reaction‐diffusion system was implemented and solved using the finite element method (comsol multiphysics 5.3, Stockholm, Sweden), the direct outputs of which were concentrations of CO_2_ and HCO_3_^−^. Net photosynthesis rate (*A*_n_) and quantum yield efficiency (ΦPSII) were then calculated.

### Plant material and growth conditions

Rice (*Oryza sativa* var. indica; IR64) plants were grown in a controlled growth chamber (Conviron; www.conviron.com) at 700 μmol m^−2^ s^−1^ photosynthetic photon flux density (PPFD) at canopy height with a 12 h : 12 h, light : dark cycle. Plants were grown at 60% humidity with a day : night temperature of 28°C : 24°C, at either ambient (480 ppm) or elevated (1000 ppm) [CO_2_]. For all assays, the middle section of mature leaf 5 was used as described (van Campen *et al*., 2016) and harvested between 21‐ and 25‐d postgermination. Plants were germinated and seedlings cultivated for 7 d in a Petri dish with 15 ml water and then transferred to 3 : 1 Levington M3 compost : vermiculite mix with 3% Osmocote^®^ Extract Standard 5–6‐month slow release fertiliser (Ipswich, UK) by volume, in 105 × 105 × 185 mm pots.

### Confocal microscopy and image analysis

Samples were stained with propidium iodide and cleared, as described (Wuyts *et al*., 2010) with the following modifications. Leaves were hand‐sectioned in transverse and longitudinal sections, then fixed and vacuum infiltrated in 3 : 1, ethanol : acetic anhydride with 0.05% Tween‐20 (v/v). Once stained, samples were cleared overnight in chloral hydrate before being mounted in water. Samples were viewed under an Olympus FV1000 using a ×40 oil objective, 543 nm laser, 555–655 nm emission. *Z*‐stacks were captured with a step size of 0.3 μm, with six biological replicates per treatment, and three to six fields of view (FOV) per replicate.

Three consecutive slices were merged in imagej (fiji v.1.51) (Schindelin *et al*., 2012); then, using multithresholding (Image Edge and Connection Thresholding plugins), individual MCs were selected to create a mask where each region of interest (ROI) represented a single cell. From the transverse images, area, maximum Feret diameter (cell length) and minimum Feret diameter (cell width) were calculated for each ROI. Mesophyll cell ROIs were created in the same way from confocal images taken in the longitudinal orientation, and cell depth was measured (maximum Feret diameter). MC volume was calculated by multiplying average MC area by average MC depth. The distance between veins and leaf thickness was measured from the transverse confocal images – the minor veins, epidermis, bulliform and mesophyll were marked in imagej, and leaf and mesophyll thickness were measured at the minor vein and the centre of the bulliform.

### Transmission electron microscopy and image analysis

Samples were prepared and analysed as described previously (van Campen *et al*., 2016). Briefly, after fixation in 3% (w/v) glutaraldehyde/0.1 M sodium cacodylate buffer and postfixation in 2% osmium tetroxide, samples were embedded in Araldite resin. Ultrathin sections (85 nm) were mounted onto 200 μm mesh copper grids, stained with uranyl acetate followed by Reynold's lead citrate and then examined using an FEI Tecnai at an accelerating voltage of 80 kV. After image capture, imagej was used for analysis, with five biological replicates per treatment, and a minimum of four FOV per replicate. Images were processed for clarity (using the Enhance Contrast function, normalised and equalised, with saturated pixels set to 5%, then smoothed using the median filter, with a radius of 10 pixels), and then parts of the sample which were overtly damaged were excluded. Masks were made of the MC walls, the plastids, the cytosol and the airspace. A whole‐cell mask was made by combining the plastid and cytosol masks. All masks were smoothed using the median filter, with a radius of 10 pixels. The perimeter and area of each mask were calculated. The proportion (%) of the cell occupied by plastid and *S*_mes_ (defined here as the proportion (%) of cell wall exposed to air) were calculated from these masks. Mesophyll cell wall thickness was measured directly from the images, at eight points per FOV. Masks were made of individual BS cells and plastids from the vascular bundle TEM images. Total BS area and average minimum Feret diameter (width) of each BS cell (thickness of BS layer) were measured, and area of plastid as a percentage of the BS area was calculated.

### MicroCT image acquisition and analysis

The method was based on that described previously (Mathers *et al*., 2018). Briefly, a 5‐mm‐diameter leaf disc was scanned using a GE Phoenix Nanotom S 180NF X‐ray microCT system (GE Sensing and Inspection Technologies GmbH, Wunstorf, Germany) at a spatial resolution of 2.75 μm. The scan consisted of the collection of 3600 projection images in ‘fast scan’ mode (sample rotates continuously), with a detector exposure time of 500 ms using X‐ray tube settings of 85 kV energy and 100 μA current. imagej was used to quantify leaf microstructure in 3D. The image analysis pipeline involved the use of a mask to remove background airspace surrounding the leaf, automated thresholding using the Li algorithm and then quantification using the particle analyser tool and BoneJ plugin (Doube *et al*., 2010). For analysis, we defined the mesophyll layer as 16.5–83.5% of the way through the leaf. This value was calculated from the confocal images of transverse sections detailed in the previous section.

### Gas exchange and chlorophyll measurements

Fluorescence and gas exchange were measured simultaneously using a Li‐Cor 6800 (Li‐Cor Inc., Lincoln, NE, USA) and an attached Multiphase Flash Fluorometer (6800‐01A). The fluorometer was set to measure *F*s *F*m′ *F*o′, with a light mode rate of 50 kHz, flash mode rate of 250 kHz and flash type: Multiphase. Measurements were taken on leaf 5, 21–25‐d postgermination, and relative humidity was maintained at *c*. 60% with the chamber flow rate set at 300 μmol s^−1^ and leaf temperature at 28°C. For *A*/*C*_i_ curves, saturating PPFD was held at 2000 μmol m^−2^ s^−1^ and the following [CO_2_]_ref_ levels were used: 400, 300, 250, 200, 150, 100, 50, 400, 500, 700, 800, 900, 1000, 1200 and 1500 ppm. Plants were held at each [CO_2_] for a minimum and maximum of 60 and 90 s for the first 7 [CO_2_] and 180–360 s for the last 7 [CO_2_] respectively. For the 8^th^ [CO_2_], plants were held until stable. For AQ curves, [CO_2_]_sample_ was maintained at 400 ppm and the following PPFD levels were used: 2000, 1800, 1600, 1500, 1200, 1000, 900, 700, 500, 400, 300, 200, 150, 100, 75, 50 and 25 μmol m^−2^ s^−1^. Plants were held at each light intensity for a minimum and maximum of 600–900 s respectively. *V*_cmax_, *J*_m_, Y(II)LL, s and Rd were calculated within *eLeaf* using these data (see Methods S1: Part B – Genetic algorithm for parameter estimation). Chlorophyll content was quantified (Porra, 2002) using 4‐mm‐diameter leaf discs harvested midphotoperiod, with five biological replicates.

## Results

### Development and implementation of the *eLeaf* model

A typical section of a rice leaf consists of a range of cell types arranged in a specific constellation, which defines the leaf histology (Fig. 1a). As a first step in modelling the cellular distribution of photosynthesis and the impact that different structural elements might have on overall leaf photosynthesis, we captured elements of this structure in a simplified model structure (Fig. 1f). In this scaffold, individual MCs are set to a range of sizes and shapes (lobe number) and then packed within a set compartmental volume. This cellular packing accommodates to levels of intercellular air space (porosity) and exposed mesophyll surface area (*S*_mes_) set by the user, informed by experimental data. In addition to measured parameters defining MC size/shape, BS area and thickness, larger leaf‐scale parameters of mesophyll and leaf thickness (at minor veins and bulliform cells) are set according to measured values. Together with interveinal distance, these parameters define the overall geometry within which MC packing occurs. In addition to these cellular and supracellular scale parameters, the model encompasses subcellular scale parameters which previous work has identified as playing a major role in defining photosynthetic performance, including the plastid proportion relative to cell size for both mesophyll and BS cells, and, related to these, the total amount of chlorophyll within a leaf segment. A final subcellular structural element included in the model is mesophyll wall thickness since this has been implicated in influencing CO_2_ diffusion from the intercellular airspace to the mesophyll cytoplasm (Ellsworth *et al*., 2018). The model allows automatic 3D reconstruction of leaf anatomy based on inputted experimental data for the parameters described above (Video S1).

In addition to the structural framework, the model comprises three modules (Fig. 1f): (1) a ‘light’ module consisting of a ray‐tracing algorithm simulating the heterogeneous internal light environment within the leaf (Xiao *et al*., 2016); (2) a ‘CO_2_’ module simulating the reaction‐diffusion process of CO_2_ from the subcellular cavities to the chloroplasts of each MC (Xiao & Zhu, 2017); and (3) a ‘metabolism’ module describing photosynthetic metabolism with the FvCB model (Farquhar *et al*., 1980), which is seeded into each modelled cell. Descriptions of each module and methods of integration are provided in Methods S1, with a list of acronyms, variables and units in Table S1.

### Model parameterisation: rice leaf structure under ambient and elevated CO_2_

To parameterise the model, we implemented a range of imaging approaches (microCT, confocal microscopy and TEM), with example images for each of these approaches from analysis of our standard rice cultivar (IR64) grown in controlled environment chambers under ambient CO_2_ (aCO_2_) shown in Fig. 1(b–e). Imaging was performed on mature leaf 5 grown under the same conditions, with all samples taken from a midportion of the leaf along both the proximal‐distal and lateral axes (excluding the midvein). Quantitative data for a range of structural parameters (listed in Table S2) were obtained using propriety software and image analysis tools (described in the Materials and Methods section), providing a comprehensive description of structure (from leaf scale through to subcellular) in a specified portion of the rice leaf at a specified stage of development.

**Fig. 2:**
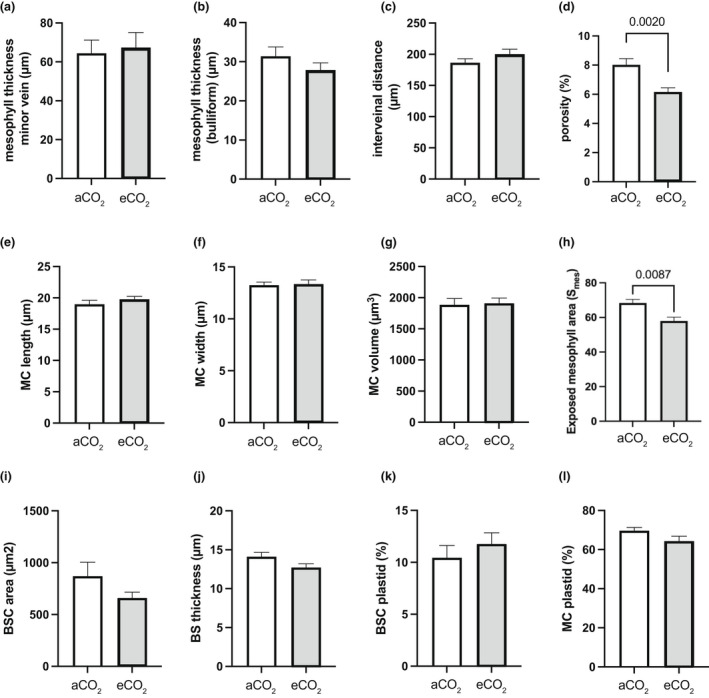
Structural properties of rice leaves grown under ambient and elevated CO_2_ levels. (a–d) whole leaf properties of mesophyll thickness at (a) minor vein; (b) bulliform cells; and (c) interveinal distance; (d) mesophyll porosity. (e–h) Mesophyll cell (MC) properties of length (e); width (f); volume (g); and exposed mesophyll surface area, *S*_mes_ (h). (i, j) Bundle sheath (BS), cell area (i) and sheath thickness (j). (k, l) Percentage of cell occupied by plastids for (k) BS cells and (l) MCs. Bars, mean values; error bars, SEM; *n* > 5, for mature leaf 5 from rice plants grown under ambient, 480 ppm CO_2_ (aCO_2_) or elevated, 1000 ppm CO_2_ (eCO_2_). Pairwise *t*‐tests were performed, with differences indicated when *P* < 0.05.

Since we had a special interest in the potential of using the *eLeaf* model to explore the influence of environmental signals on leaf structure and photosynthesis, we also performed our analysis of leaf structure on IR64 plants grown under conditions of elevated levels of 1000 ppm CO_2_ (eCO_2_), a factor of broad interest in terms of future climate (Sanz‐Saez *et al*., 2017). The results of these analyses are shown in Fig. 2.

Comparing plants grown under aCO_2_ or eCO_2_ at the leaf scale, there was no significant difference in mesophyll thickness at the positions either of the minor veins (Fig. 2a) or the bulliform cells (Fig. 2b), and there was no significant difference in the distance between minor veins (Fig. 2c). However, when mesophyll porosity was analysed, there was a significant decrease in leaves, which developed under eCO_2_ (Fig. 2d). Such a change could arise by a number of cellular routes involving MC size, shape and separation (Lundgren & Fleming, 2020). To explore these possibilities, we examined various parameters related to leaf cellular architecture. In terms of mesophyll length, width and volume, there was no significant difference between cells from leaves grown in aCO_2_ or eCO_2_ (Fig. 2e–g); however, comparison of exposed mesophyll surface area (*S*_mes_) indicated a significant decrease in leaves from plants grown under eCO_2_ (Fig. 2h). An increase in tissue per volume with no significant change in cell size suggests that the decrease in mesophyll porosity observed in Fig. 2(d) occurred via an increase in MC number, with a closer packing of the cells resulting in the observed decrease in *S*_mes_ (Fig. 2h). We also parameterised the *eLeaf* model with values for BS area (Fig. 2i) and thickness (Fig. 2j), with comparison of these features revealing no significant difference between aCO_2_ and eCO_2_ grown leaves. At the subcellular scale, although there was a tendency for an increased percentage of chloroplast occupation of BS cells in leaves grown under eCO_2_ (Fig. 2k), this was not statistically significant. There was a slight decrease in the percentage occupation of the MCs by chloroplasts in the eCO_2_ leaves, but this was also found to be not statistically significant (Fig. 2l), suggesting (as backed up by observation of TEM images) that the plasma membrane of the MCs in the eCO_2_ plants was highly covered by chloroplasts, as is normal for rice MCs (Sage & Sage, 2009).

### Model validation

The mean values obtained for the various structural parameters described in Fig. 2 for leaves grown in aCO_2_ were used as inputs to define the initial conditions for the *eLeaf* model. In addition, a range of physiological and biochemical parameters were used to define model conditions. These are listed in Table S3 and were either derived from combined fluorescence gas exchange analysis of tissue at the same developmental stage, biochemical analysis or, for some constants, taken as accepted values from the literature. The *eLeaf* model was then run and output values obtained for assimilation rate, *A*, and effective quantum yield of photosynthesis, ΦPSII. While the input anatomical parameters constrain the modelled cell packing, they do not define it completely. Thus, a given set of cellular structural inputs can result in a range of possible modelled 3D geometries, each with a slightly different distribution of tissue/air space, with potential consequences for modelled CO_2_ and light distribution patterns. To account for this, five model replicates were reconstructed for each set of anatomical input parameters and the model was run to give five independent sets of model outputs for either assimilation rate (*A*) or ΦPSII at any range of *C*_i_ and irradiance.

**Fig. 3:**
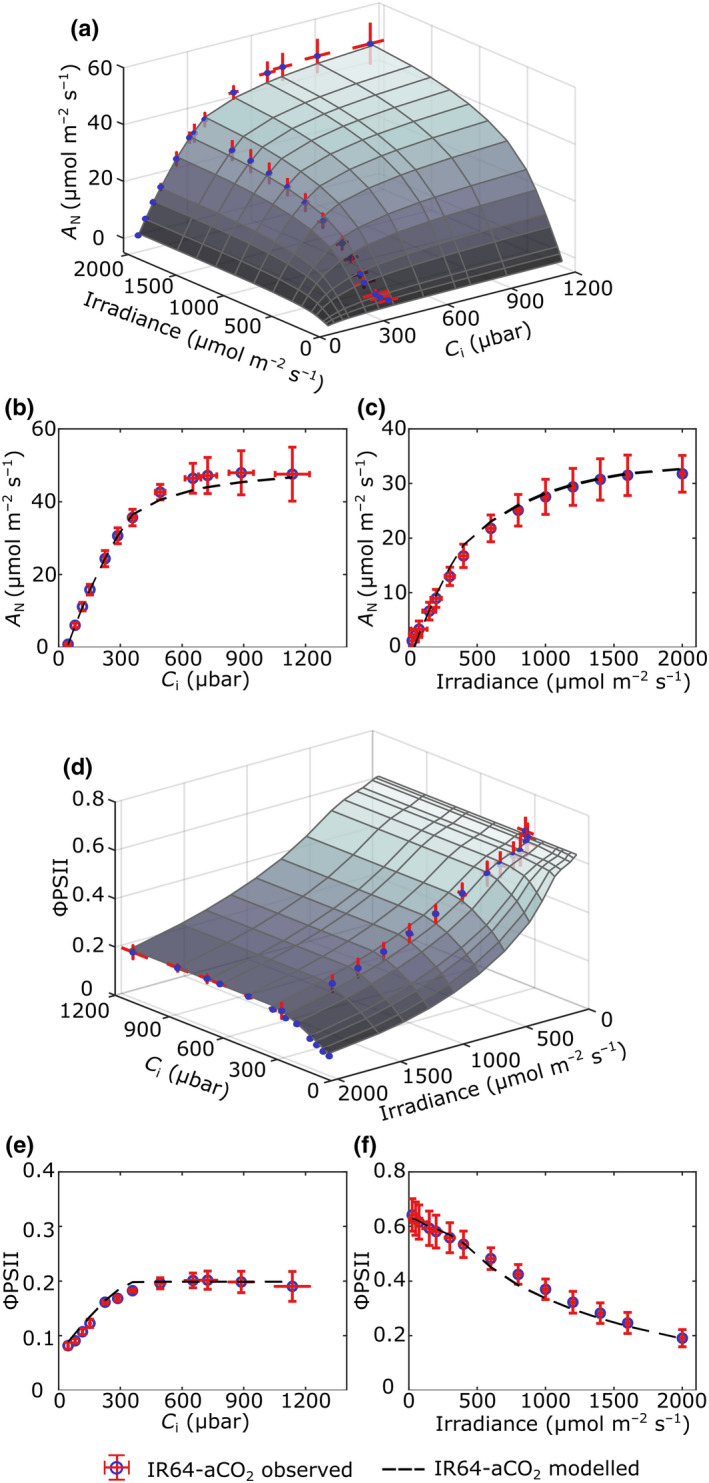
Validation of the *eLeaf* model against measured photosynthesis for rice leaves grown under ambient CO_2_. Surface plots of the modelled net photosynthetic CO_2_ uptake rates (*A*_N_) (a) and quantum yield of photosystem II (Φ_PSII_) (d) under different light and CO_2_ levels for plants grown under aCO_2_. Measured curves at constant high irradiance and constant ambient CO_2_ levels are superimposed in each plot (blue points with error bars in red). (b, c) Comparison of measured and modelled *A*/*C*_i_ (b) and *A*/*Q* curves (c) for leaves grown under aCO_2_. Measured values are shown as blue circles, with error bars (SD) in red. Modelled curves (the mean of five replicate runs) are shown as a black dashed line. (e, f) Comparison of measured and modelled ΦPSII/*C*_i_ (e) and Φ_PSII_/*Q* curves (f) for leaves grown under aCO_2_. Symbols as in (b).

Modelled mean output values are shown in Fig. 3(a) as a surface plot linking assimilation rate (*A*), irradiance and *C*_i_ for leaf structure under aCO_2_ conditions. Exemplar *AC*_i_ (Fig. 3b) and AQ (Fig. 3c) curves are shown to allow comparison of the modelled outputs (dashed lines) with the experimentally measured values (open circles) obtained by infrared gas analysis. Considering the surface plot in Fig. 3(a), as expected, a plateau of *A*_max_ is obtained at high *C*_i_ and irradiance of slightly above 40 μmol m^−2^ s^−1^. Values of *A* drop off rapidly at *C*_i_ values below *c*. 300 μbar and irradiance below 750 μmol m^−2^ s^−1^ respectively. Comparing the modelled outputs and measured values, at higher *C*_i_ values the modelled values slightly underestimate the observed values of *A* (Fig. 3b) and in the early phase of the AQ curve (lower irradiance) the modelled values slightly overestimate the observed values (Fig. 3c). Considering ΦPSII, the surface plot in Fig. 3(d) demonstrates the modelled relationship of ΦPSII with *C*_i_ and irradiance, with exemplar ΦPSII/*C*_i_ and ΦPSII/Q curves shown in Fig. 3(e,f) respectively. ΦPSII reaches a minimum value of approximately 0.1 at high irradiance and is relatively unresponsive to *C*_i_ until values fall below *c*. 300 μbar (Fig. 3d). Comparing the modelled outputs and measured values, there is a slight overestimate of ΦPSII at lower *C*_i_ values (Fig. 3e) and an underestimate at mid–high irradiance levels (Fig. 3f). However, overall, the modelled and observed curves show a strong similarity, for both *A* and ΦPSII, suggesting that the *eLeaf* model successfully captured photosynthetic performance when parameterised with data describing leaves grown under aCO_2_.

**Fig. 4:**
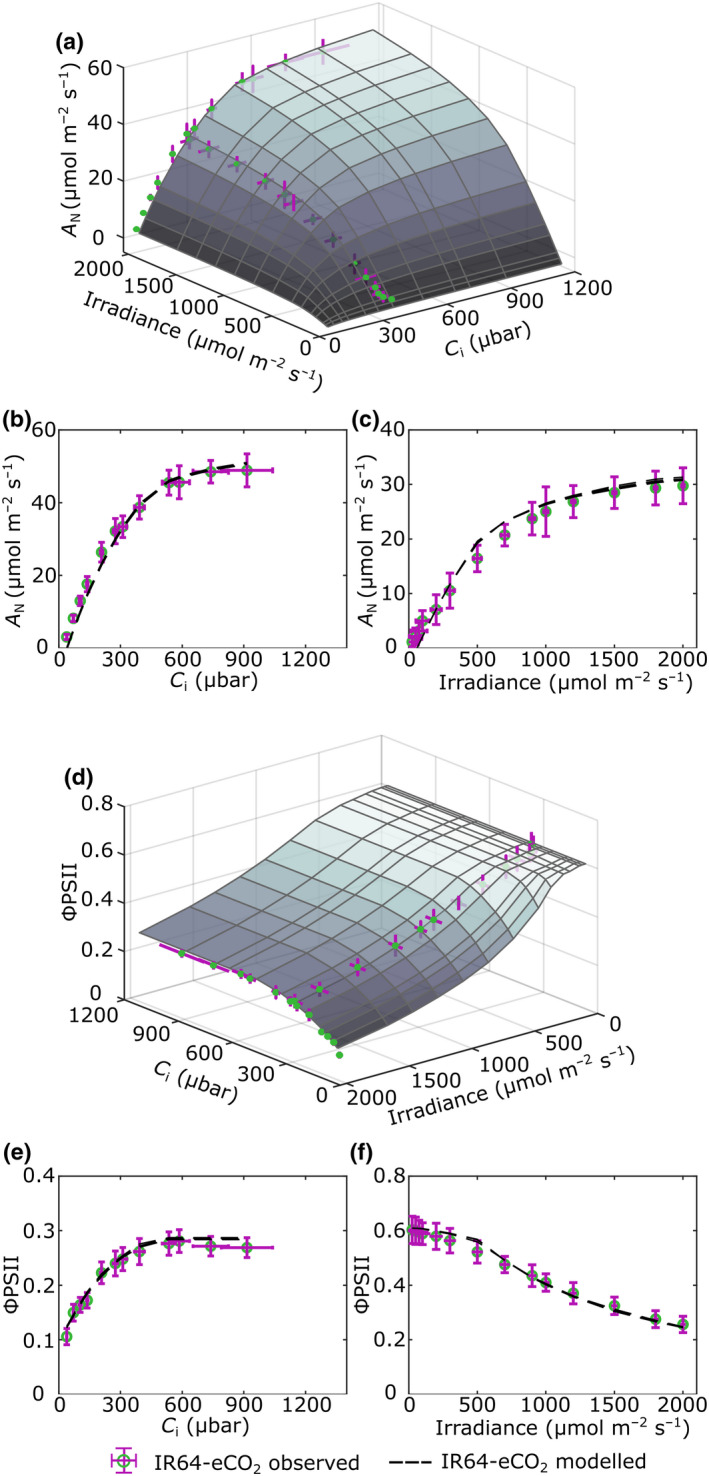
Validation of the *eLeaf* model against measured photosynthesis for rice leaves grown under elevated CO_2_. Surface plots of the modelled net photosynthetic CO_2_ uptake rates (*A*_N_) (a) and quantum yield of photosystem II (Φ_PSII_) (d) under different light and CO_2_ levels for plants grown under eCO_2_. Measured curves at constant high irradiance and constant ambient CO_2_ levels are superimposed in each plot (blue points with error bars in purple). (b, c) Comparison of measured and modelled *A*/*C*_i_ (b) and *A*/*Q* curves (c) for leaves grown under aCO_2_. Measured values are shown as blue circles, with error bars (SD) in purple. Modelled curves (the mean of five replicate runs) are shown as a black dashed line. (e, f) Comparison of measured and modelled Φ_PSII_/*C*_i_ (e) and Φ_PSII_/*Q* curves (f) for leaves grown under eCO_2_. Symbols as in (b).

When mean values for structural elements measured in leaves grown under eCO_2_ conditions were used to parameterise the *eLeaf* model, again a close fit between modelled and observed values was obtained. Fig. 4(a) shows a surface plot relating *A*, irradiance and *C*_i_ for an eCO_2_ leaf structure. A modelled peak value of *A* is obtained at high *C*_i_ and irradiance, with *A* falling steeply below *C*_i_ values of *c*. 500 μbar. Response to irradiance is more gradual, with a decline in *A* becoming more apparent below 1000 μmol m^−2^ s^−1^. Comparing the modelled and measured outputs, the modelled *A*/*C*_i_ curves closely resemble the observed values (Fig. 4b). In the AQ curve, the modelled values provide a slight overestimate of the measured values, particularly at mid–high irradiance values (Fig. 4c). With respect to ΦPSII, a modelled minimum value of slightly above 0.1 is recorded at high irradiance for most values of *C*_i_, with ΦPSII falling away at *C*_i_ values below *c*. 300 μmol m^−2^ s^−1^ except at low irradiance levels (Fig. 4d). Considering the modelled and measured output values, *eLeaf* slightly overestimates ΦPSII with respect to *C*_i_ (Fig. 4e), but provides a good alignment with ΦPSII response to irradiance, with a slight overestimate at very low values of irradiance (Fig. 4f). Despite these individual discrepancies, the modelled and observed curves show a strong similarity, for both *A* and ΦPSII, suggesting that the *eLeaf* model has successfully captured photosynthetic performance when parameterised with data describing leaves grown under eCO_2_. Taken together, the data shown in Figs 3 and 4 indicate that *eLeaf* does successfully capture elements of photosynthetic performance for a range of external values of *C*_i_ and irradiance for both aCO_2_ and eCO_2_‐associated leaf structures.

### Analysis of the relative contribution of anatomy and metabolism to leaf photosynthetic performance

**Fig. 5:**
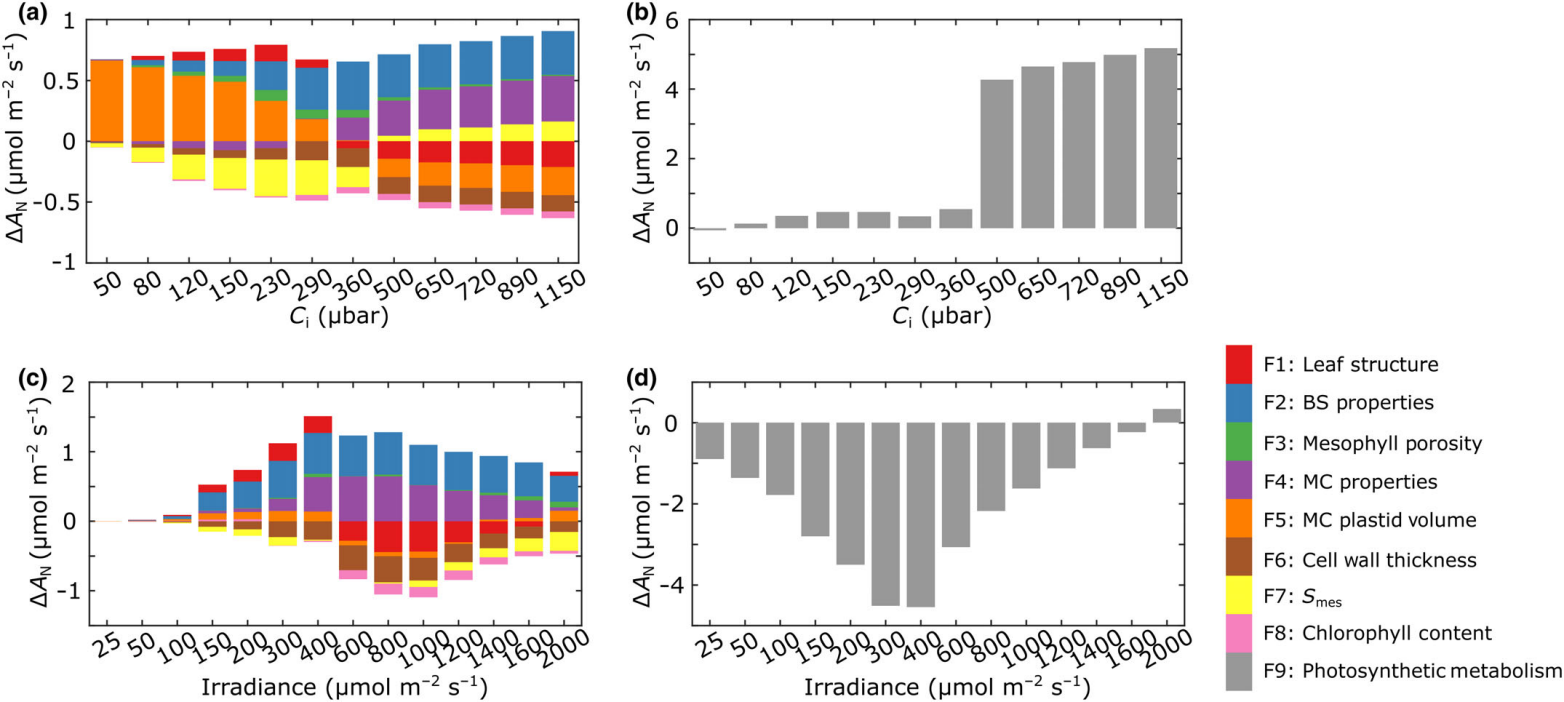
Interrogation of the *eLeaf* model reveals the relative contribution of structural and metabolic parameters to altered carbon assimilation rate under elevated CO_2_. The contribution of structural parameters (a, c) and photosynthetic metabolism (b, d) to the altered leaf assimilation rate (Δ*A*_N_) (either positive or negative) of rice leaves grown under elevated CO_2_ according to a range of imposed *C*_i_ values (a, b) or irradiance levels (c, d). The values are given for each factor (*F*_1–9_) within the *eLeaf* model, as indicated by the colour legend.

As indicated in the Introduction section, an advantage of the mechanistic modelling approach is that, once validated, it is possible to explore the model to identify the relative contribution that different parameters make to particular outputs under specific sets of conditions. To investigate the potential contribution of the different structural parameters described in Fig. 2 to photosynthetic performance under aCO_2_ and eCO_2_ conditions, we performed an analysis whereby the *eLeaf* parameters were placed into nine categories, *F*_1–9_ (listed in Tables S2, S3). *F*_1–7_ describe structural parameters, *F*_8_ describes chlorophyll content, and *F*_9_ encompasses the metabolic processes, which underpin the *eLeaf* model within each virtual cell. By running the *eLeaf* model under the input group values (*F*_1–9_) for aCO_2_ but substituting a single parameter group input value obtained under eCO_2_ (*F*_*x*_), it was possible to estimate the contribution to total assimilation rate of the eCO_2_ value for each parameter group *F*_*x*_. If the modelled output value for *A* increased, then the structural changes observed under eCO_2_ were having a positive effect on *A*, whereas if the modelled output value for *A* decreased, then the structural changes observed under eCO_2_ were having a negative effect on *A*. We did this sequentially for each parameter group (*F*_1–9_), with the results shown in Fig. 5. These graphs show the change in assimilation rate, Δ*A* (either positive or negative), that each parameter group, *F*_1–9_, makes under eCO_2_ conditions. This has been calculated for a range of *C*_i_ (Fig. 5a,b) and a range of irradiance values (Fig. 5c,d), with the absolute contribution to assimilation rate (positive or negative) made by structural elements shown in Fig. 5(a,c) and the contribution made by changes in photosynthetic metabolism shown in Fig. 5(b,d).

Considering the influence of structural elements as *C*_i_ changes (Fig. 5a), at higher CO_2_ levels, the small experimentally measured changes in MC and BS size (*F*_2_, *F*_4_) were modelled to have a positive outcome on assimilation rate, as was *S*_mes_ (*F*_7_). However, these positive outcomes were to some extent negated by the decrease in assimilation rate related to the observed changes in mesophyll plastid volume (*F*_5_) and cell wall thickness (*F*_6_) under eCO_2_. It is noticeable that under CO_2_ levels above ambient mesophyll plastid volume had a negative outcome on *A*, whereas at lower *C*_i_ values it was modelled to have a positive effect, with *S*_mes_ showing the opposite switch (beneficial under elevated *C*_i_, detrimental with respect to *A* under elevated *C*_i_).

No single structural parameter makes an overwhelming contribution to altered carbon assimilation rate, though it is interesting to note that the parameter which showed the most striking experimentally measured change (mesophyll porosity, *F*_3_; Fig. 2d) is modelled to have little or no impact on assimilation rate. Under relatively high CO_2_ levels, the modelled increase in assimilation rate can be largely attributed to shifts in photosynthetic metabolism, *F*_9_, associated with growth under eCO_2_ (Fig. 5b).

With respect to how the structural changes observed in leaves grown under high CO_2_ influence assimilation response to irradiance level (Fig. 5c), again both positive and negative effects on assimilation rate were modelled. The measured changes in MC and BS properties (*F*_2_, *F*_4_) have the major positive effect on assimilation rate, with leaf structure (*F*_1_), cell wall thickness (*F*_6_) and (particularly at higher irradiance) *S*_mes_ (*F*_7_) having a negative outcome on assimilation rate. As with the response to *C*_i_, the major (negative) outcome on assimilation rate is related to shifts in photosynthetic metabolism (*F*_9_) associated with growth in eCO_2_ (Fig. 5d), but this is most significant at lower irradiance levels (below 1000 μmol m^−2^ s^−1^). As with the response to *C*_i_, mesophyll porosity (*F*_3_) is modelled to have little or no influence on carbon assimilation rate response to irradiance.

### Exploring the role of mesophyll porosity, *S*_mes_ and MC shape in photosynthesis

**Fig. 6:**
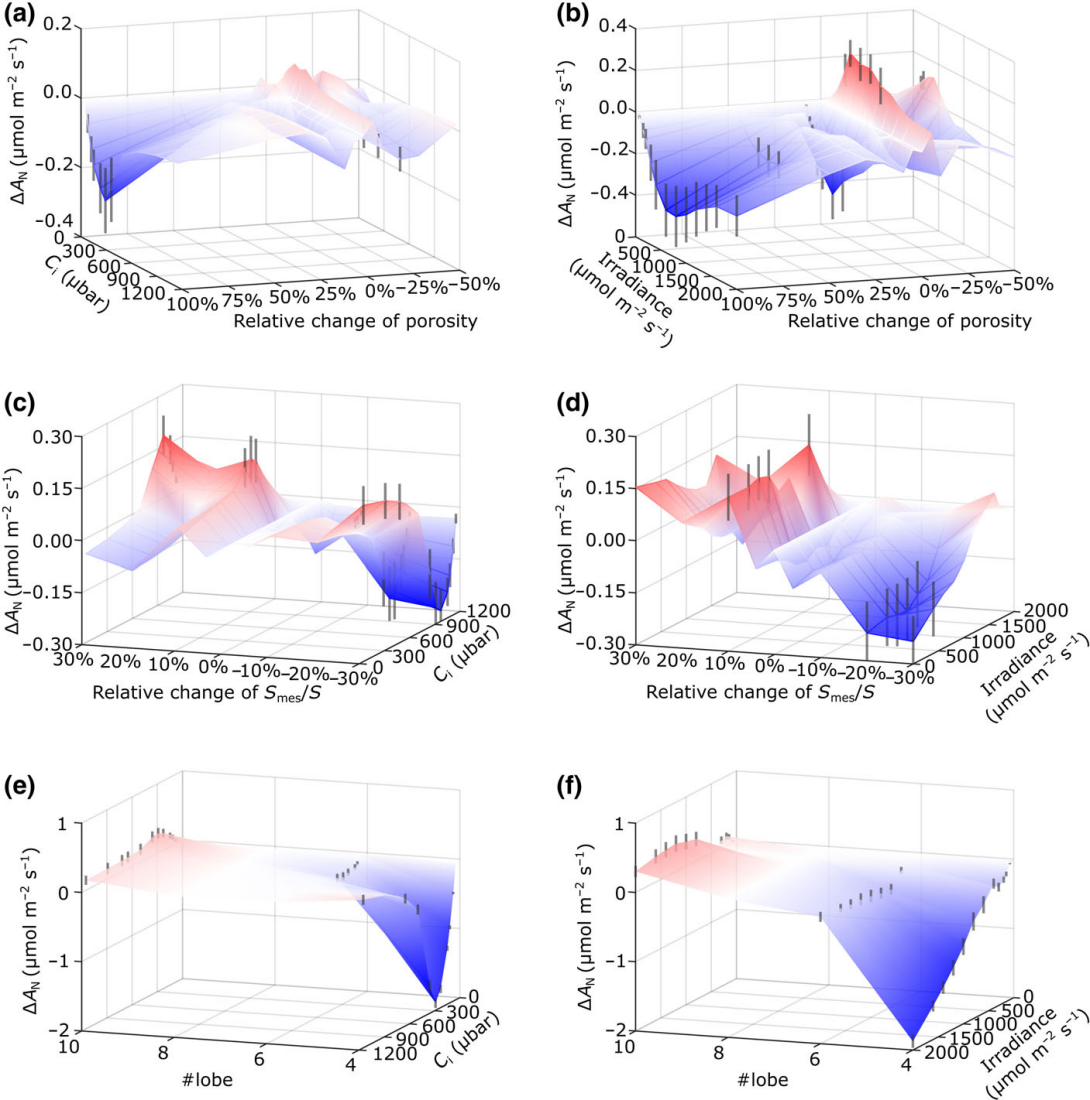
Exploration of parameter space identifies the changes in mesophyll porosity, exposed mesophyll cell (MC) area and cell lobing required to significantly alter leaf assimilation rate. (a, b) Surface plots of changes in assimilation rate (Δ*A*_N_) in response to change in mesophyll porosity for a range of *C*_i_ values (a) or irradiance (b), with other model parameters set and maintained according to measured values from rice leaves grown under aCO_2_. (c, d) Surface plots of changes in assimilation rate (Δ*A*_N_) in response to change in relative change in exposed MC area (*S*_mes_/*S*) for a range of *C*_i_ values (c) or irradiance (d), with other model parameters set and maintained according to measured values from leaves grown under aCO_2_. (e, f) Surface plots of changes in assimilation rate (Δ*A*_N_) in response to change in MC lobe number (# lobe) for a range of *C*_i_ values (e) or irradiance (f), with other model parameters set and maintained according to measured values from leaves grown under aCO_2_. Red shading indicates an increase in assimilation rate and blue a decrease. Vertical black lines (indicating the range of modelled values) are shown only for those shifts calculated to lead to a significant change (ANOVA; *P* < 0.05) in assimilation rate compared with control values.

An advantage of the mechanistic modelling approach is that once potentially interesting parameters are identified, it is possible to explore the influence of that parameter on the model output, via either an analytical or empirical approach. From the *eLeaf* model outputs described above, one result of interest was the apparent lack of influence of mesophyll porosity (Fig. 5a,b), despite the fact that this was one of the structural parameters that showed a statistically significant change after growth of the plants in eCO_2_ (Fig. 2d). To explore this observation further, we imposed a range of porosity values within the *eLeaf* framework under aCO_2_ conditions, keeping other values as constant as possible within the constraints imposed by the modelling boundaries. These data (Fig. 6a,b) indicated that, indeed, mesophyll porosity values could vary over a relatively large range before any major shift in the output of carbon assimilation rate. Relative shifts in porosity of over 50–75% were generally required for any negative outcome, with some positive influence on assimilation rate being observed only under low irradiance (< 1000 μmol m^−1^ s^−2^) at decreased porosity values of *c*. 20%.

With respect to exposed *S*_mes_, we explored the influence of the shift in this parameter calculated on a per cell surface area (i.e. exposed cell area relative to the total surface area of each MC, *S*_mes_/*S*). These results (Fig. 6c,d) indicated that relatively small increases in this parameter (5%) would have some positive outcome on assimilation rate under most irradiance levels but only under conditions lower than ambient *C*_i_. A significant negative outcome was modelled under conditions of high *C*_i_ and high irradiance when *S*_mes_/*S* was decreased beyond 10–15%. The *S*_mes_ : *S* ratio is likely to be influenced by the shape of the cell, in particular the degree of lobing, a distinctive feature of the rice mesophyll. We therefore empirically explored the influence of this parameter on the relationship of carbon assimilation rate to *C*_i_ and irradiance. The range of cell shapes imposed is shown in Fig. S1, with the resulting cellular architectures and the spatial outcome on light absorption and internal CO_2_ levels displayed in Fig. S2. When lobe number was varied within the model between 4 and 10 (maintaining the model with otherwise unchanged aCO_2_ or eCO_2_ structural and metabolic parameters, within model constraints), there was very little influence on the relationship of assimilation rate and *C*_i_ until a lobe number of 4 was reached, at which point assimilation rate decreased markedly (Fig. 6e). Interestingly, a similar situation was observed in the assimilation/irradiance relationship, with a sharp reduction in assimilation at high irradiance observed only when lobe number was decreased below 6 (Fig. 6f).

## Discussion

### eLeaf enables dissection of the mechanistic basis of photosynthetic variation in leaves

The biochemical and ultrastructural elements of photosynthesis are distributed in space via a highly ordered yet complex physical scaffold: the leaf and its component cells. Although it has long been recognised that variation in leaf and cellular architecture has a significant impact on photosynthetic performance (Terashima *et al*., 2011; Lundgren & Fleming, 2020), assigning quantitative values to the various parameters involved in a mechanistic context has proved challenging (Earles *et al*., 2019). In this paper, we report on the development and implementation of a model, which facilitates this for a major staple crop, rice. Combining a range of imaging techniques (capturing aspects of rice leaf structure at scales from the whole leaf to the subcellular) with a custom‐built algorithm for reconstruction, we were able to convert experimental measurements into representative 3D models, which were used as a scaffold for modelling the spatial distribution of light, CO_2_ and the photosynthetic activity of individual cells within a virtual leaf. Integration of the outputs of these virtual cells enabled us to simulate photosynthetic performance at the whole organ level.

As with all models, *eLeaf* depends upon a number of assumptions. For example, the measured amount of chlorophyll is assumed to be uniformly distributed across all cells, which probably does not reflect reality (Vogelmann & Evans, 2002; Borsuk & Brodersen, 2019). Similar assumptions are involved in the estimated profiles of light absorption, *V*_cmax_ and *J*_m_, which are difficult to experimentally validate, although investigations using microscopic fibre optic probes or imaging chlorophyll fluorescence in transverse sections with illumination via the adaxial surface present opportunities to address these issues (Takahashi *et al*., 1994; Oguchi *et al*., 2011). Future areas for model refinement also include the potential inclusion of triose phosphate utilisation limitation to the model (Harley & Sharkey, 1991; Sage, 1994). The 3D construction algorithm is also an area for possible future improvement. In particular, the porosity parameter implemented in *eLeaf* does not incorporate related features such as tortuosity or connectivity, features which theoretically allow the same absolute value of porosity to have distinct influences on CO_2_ diffusion and water evaporation (Earles *et al*., 2019).

Despite these necessary simplifications, comparison of modelled and observed outputs (Figs 3, 4) demonstrated a close similarity over a range of *C*_i_ and irradiance values, indicating that the *eLeaf* model does successfully capture core elements of leaf photosynthetic performance. This represents an advance on previous models of photosynthesis. Compared with earlier efforts, this is the first model which enables not only a realistic presentation of 3D leaf structural and biochemical properties for a given leaf (instead of a generic or hypothetical leaf) but also *in silico* experiments to examine the impact of changing particular properties on leaf photosynthesis (Zhu *et al*., 2013; Ho *et al*., 2016; Xiao & Zhu, 2017). It should also be noted that although rice is the focus here, the modular programming adopted in *eLeaf* makes lots of the code potentially reusable when modelling leaf structure in other species. Providing data for a new 3D reconstruction module are obtained, then it should be possible to perform similar analyses for leaves with comparable geometric complexity as the rice leaves reported here. The model provides a theoretical framework to quantitatively evaluate the contribution of different biochemical and structural features–factors to leaf photosynthetic performance under different environmental conditions and, moreover, to investigate the potential importance of specific traits by exploring the relevant parameter space, as discussed below.

### The importance of leaf structure in photosynthetic performance

As implicitly assumed in many current projects aiming to engineer photosynthetic metabolism for greater efficiency (Long *et al*., 2015; Walker *et al*., 2016; South *et al*., 2019; Ermakova *et al*., 2020), our analysis indicates that metabolism plays a major role in dictating carbon assimilation rate. This is particularly true under conditions of elevated *C*_i_ where our modelling suggests it contributes up to 90% of the increased assimilation response, with the remaining 10% related to a range of structural components. The *eLeaf* model thus enables a quantitation of the contribution of various factors to photosynthetic performance which previous models have not provided. The *eLeaf* model also helps reveal the environment‐dependent nature of these contributions and the complexity arising from their interactions, interdependencies which modelling at other scales is also revealing (Wu *et al*., 2019). For example, although the metabolic shifts associated with growth in eCO_2_ make a major positive contribution under conditions of high CO_2_, if that is combined with (for rice) relatively low irradiance levels, then the same metabolic shifts can act in a negative fashion to limit the achievable increases in assimilation rate. Of course, there is the caveat that the underlying assumptions of the model mean that the precise output values must be taken as indicative rather than absolute, but the *eLeaf* model demonstrates how it is possible to relatively rapidly gain insights into the complex relationship of photosynthetic structure/function/environment, hopefully helping to focus future effort in experimental investigations.

Considering the influence of the different leaf structural components in the model on assimilation rate, our analysis provides a quantitative estimate of their absolute and relative contributions. No single structural parameter has an overwhelmingly strong influence on assimilation rate, with some structural changes actually leading to a negative outcome on carbon assimilation rate, with the outcome dependent on the prevalent *C*_i_ and light environment. Removal of these structural brakes might be a novel approach to maximising assimilation rate. One striking observation from our work was that some parameters showing significant measured shifts after growth in eCO_2_ had essentially no outcome on modelled photosynthetic performance under most conditions. In particular, our results indicated that large changes in mesophyll porosity could be accommodated by the leaf, with essentially no effect (positive or negative) on assimilation rate over a very wide range of *C*_i_ and irradiance levels. This result may appear at first sight surprising, but there are a number of strands of evidence in the literature indicating that leaves can show an extraordinary plasticity in relative amount of tissue per volume, with some leaves with extremely low cell mass per volume still managing to photosynthesise and grow (Gonzalez‐Bayon *et al*., 2006; Whitewoods, 2021). Our results provide a theoretical underpinning to explain and support these observations. Bearing in mind the carbon and nitrogen costs involved in generating photosynthetic tissue, our results align with ideas that crops with increased leaf porosity may still be able to maintain a good level of photosynthetic assimilation rate with less investment in leaf structure (Ort *et al*., 2015).

Another factor of specific relevance to rice is the extreme lobing of MCs, which is thought to provide increased surface area per volume, allowing for the alignment of chloroplasts along this surface and, consequently, increased capacity for gas flux from the leaf airspace to the site of carbon assimilation within the chloroplasts (Sage & Sage, 2009). According to our model, exposed *S*_mes_ does contribute in a positive fashion to increased assimilation under higher levels of *C*_i_ but may actually have a negative influence under high irradiance. Exploiting the capacity of the modelling approach to impose changes in the degree of MC separation (*S*_mes_/*S*) and in the degree of cell lobing time‐consuming to engineer in the laboratory, we found that the relationship of assimilation rate to *C*_i_ was sensitive to *S*_mes_/*S*, but that at higher *C*_i_ values, the gains were very limited. Interestingly, lobe number had limited influence on the relationship of assimilation to *C*_i_ or irradiance until lobe number decreased to 4, at which point a dramatic decrease occurred. Why assimilation rate should decrease under high irradiance for leaves filled with MCs with low lobe number is unclear. One possibility is that, in addition to a role in optimising gas exchange via its influence on *S*_mes_/*S*, lobing influences cell packing (Wilson *et al*., 2021) and, thus, the distribution of chloroplasts across 3D space, influencing light absorption within the leaf. Thus the data provide an example where the modelling platform allows the identification of intriguing/unexpected output scenarios, which can then be the focus for experimental testing. The results also suggest that efforts to alter the number of MCs between veins to engineer improved photosynthesis (Ermakova *et al*., 2020) should not *a priori* have any detrimental outcome on the underlying process of photosynthesis provided lobe number is maintained.

In conclusion, the *eLeaf* model provides the basis for future experimental work to explore the importance of specific structural elements of the rice leaf and provides a theoretical context for efforts to engineer improved photosynthetic performance in rice. The combined modelling and experimental framework also provides the foundation for a similar approach in other major crops.

## Competing interests

None declared.

## Author contributions

YX, JS, CH, MF‐S, AM, RT, AB, HJ, TC and XC performed the experiments. CS and SJM supervised the CT analysis and interpretation. CPO advised on fluorescence‐linked gas exchange analysis and interpretation. YX, JS, CH, NY, X‐GZ and AJF interpreted the results and wrote the paper, with contributions from all authors. AJF and X‐GZ designed the study and led the project. YX, JS and CH contributed equally to this work.

## Supporting information


**Fig. S1** Varying lobe number in the *eLeaf* model.
**Fig. S2** Sensitivity analysis to mesophyll cell lobing.
**Methods S1** The *eLeaf* model.
**Table S1** Acronyms, definitions, variables and units used.
**Table S2** Structural parameters for the *eLeaf* model.
**Table S3** Metabolic and physiology parameters for the *eLeaf* model.

**Video S1** Generation of the *eLeaf* model.
Please note: Wiley is not responsible for the content or functionality of any Supporting Information supplied by the authors. Any queries (other than missing material) should be directed to the *New Phytologist* Central Office.

## Data Availability

The experimental data (image and gas exchange data) are available on request from the corresponding author. All code is available at GitHub with instructions on use provided in Supporting Information.
