# Supplementary material for: Defining the scope for altering rice leaf anatomy to improve photosynthesis: a modelling approach
Source: New Phytol. 2022 Nov 25;237(2):441–53. doi: 10.1111/nph.18564 (PMC10099902; doi:10.1111/nph.18564)
Supplement: Supplementary file 1 — Fig. S1 Varying lobe number in the eLeaf model. Fig. S2 Sensitivity analysis to mesophyll cell lobing. Methods S1 The eLeaf model. Table S1 Acronyms, definitions, variables and units used. Table S2 Structural parameters for the eLeaf model. Table S3 Metabolic and physiology parameters for the eLeaf model. [file NPH-237-441-s002.pdf]

New Phytologist Supporting Information

Article title: Defining the scope for altering rice leaf anatomy to improve photosynthesis: A modelling approach

Authors: Xiao, Yi; Sloan, Jennifer; Hepworth, Chris; Fradera-Soler, Marc; Mathers, Andrew; Thorley, Rachel; Baillie, Alice; Jones, Hannah; Chang, Tiangen; Chen, Xingyuan; Yaapra, Naxmin; Osborne, Colin; Sturrock, Craig; Mooney, Sacha; Fleming, Andrew; Zhu, Xin-Guang

Article acceptance date: 4th October 2022

SI Methods: THE eLEAF MODEL

THEORY GUIDE FOR THE eLEAF MODEL

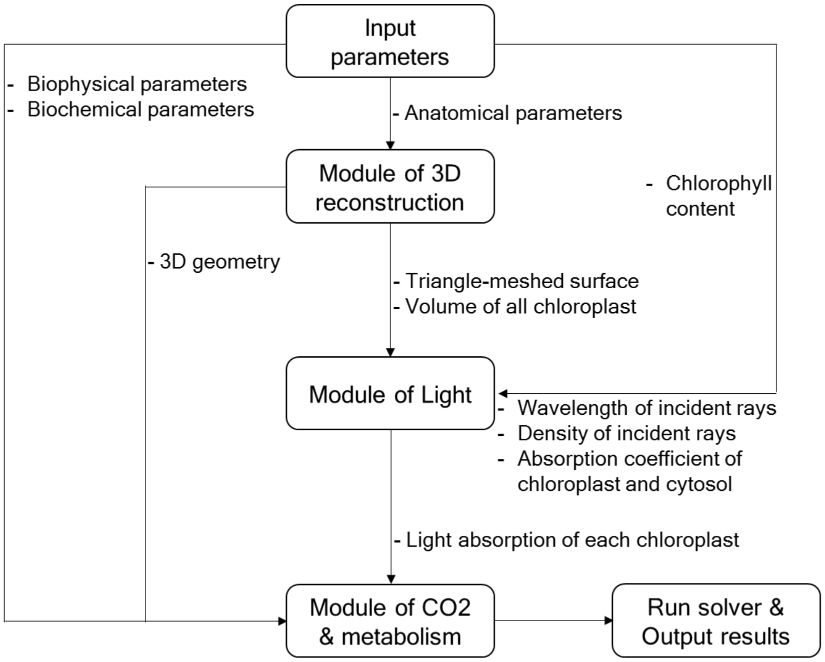

Workflow of *eLeaf*, and inputs & outputs between modules.

- Module of 3D reconstruction

In order to reconstruct a 3D leaf geometry with exactly the same anatomical features as the measured values (see Table 1), a delicate design of cell shape and cellular structure was developed, as shown in the accompanying diagram of a mesophyll cell with six lobes, where the structure is controlled by parameters a-l and number of lobes of the mesophyll cell.

- 'a' controls the thickness of cytosol between chloroplast layer and cell wall
- 'b' controls the thickness of chloroplast layer
- 'c' controls the position of mitochondria
- 'd' controls the size of spheric mitochondria
- 'e' and 'f' control the size of vacuole

- ‘g’ and ‘h’ are used to control S_m, i.e. contact area of mesophyll cell surface to the intercellular air space
- ‘j’, ‘k’ and ‘l’ are the length, width and thickness of mesophyll cells

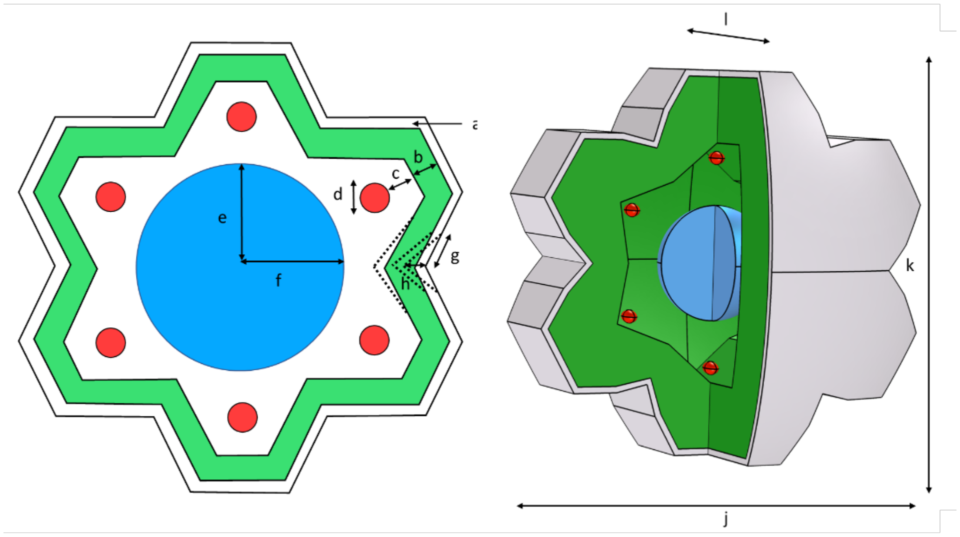

Design of a mesophyll cell with six lobes in *eLeaf* model.

Then an automatic pipeline was developed to match the measured anatomical features one by one.

1. First, at the cellular level, depth of mesophyll cell (MC) was calculated based on its length, width, and volume.
2. Parameter ‘g’ in Fig. S2 was determined based on measured Sm.
3. Then thickness of chloroplast layers was calculated to match the percentage of plastid area in MCs.
4. Since the measured number of lobe equals to four, the maximum number of lobe allowed for IR64_aCO2 model and IR64_eCO2 model was set to be six.
5. Next, a frame of leaf unit was built based on leaf/mesophyll thickness at minor vein and at bulliform cells, and also shape and size of bundle sheath cells. Then, we started to fill in the region of mesophyll with MCs. (see animation clip - ‘Match mesophyll porosity’).
6. MSCs with different number of lobe were filled into the space in a compact way to reach a leaf porosity value closest to the measured one. Then thickness of the modeled leaf unit was adjusted to match the porosity exactly, considering the laminar structure of rice leaf in the longitudinal direction

- Module of internal light propagation

With the reconstructed 3D leaf, triangle surface meshes were generated for all cells and cellular structures including chloroplast layer and vacuole. Mitochondria were neglected in current ray tracing to reduce the computational complexity. The same ray tracing algorithm as Xiao *et al.*, 2016 was applied here to simulate the light propagation inside leaf and predict light absorptance of each chloroplast in both mesophyll cells and bundle sheath cells.

To compare with the measured physiological data from Licor 6800 (LI-COR Inc., Lincoln, NE, USA), the light source was set to be collimated light with 90% red light (625nm) and 10% blue light (475nm). Special boundary conditions were assigned to the front, back, left, and right surfaces of the leaf unit, so that rays hit these boundaries would be reflected back into the leaf unit. In another word, only rays hit the upper or lower boundary would be counted as total reflectance and transmittance respectively.

Reflection and refraction of light on the interface of two substances were calculated based on Fresnel equations. Meanwhile, light absorption inside certain substance was calculated from Beers-Lambert's law. Same refractive indexes and absorption coefficients of cell walls, cytosol, chloroplasts, and vacuoles as Xiao *et al.,* 2016 were adopted. Absorption coefficient of chloroplast is also linear to the chlorophyll concentration. Here by assuming the chlorophyll concentration distributes uniformly across the leaf, we can calculate it from the measured chlorophyll content per leaf area and the information about chloroplast volume based on the reconstructed 3D leaf geometry.

- Module of CO_2 reaction-diffusion and photosynthetic metabolism

A constant CO_2 concentration ([CO_2]) was set in the substomatal cavity in the 3D geometry as part of the boundary condition. For the purposes of *eLeaf*, the rest of the epidermis was assumed to be impermeable to CO_2. In the intercellular air space, CO_2 molecules move following the diffusion equation. On the outer surface of the cell wall, [CO_2] in the gaseous phase was converted to [CO_2] in the liquid phase following Henry's Law.

The internal surface representing the cell wall and plasma membrane was modelled as a thin diffusion barrier with a given permeability for CO_2,

$$n \cdot (-D\nabla C) = P_{CO2}(C_1 - C_2) \quad \text{(S1)}$$

where n is the normal vector of the surface, therefore the left-hand side of the equation is the diffusive flux. P_{CO2} (m s^{-1}) is the permeability of the boundary to CO_2, and C_1 and C_2 (mol m^{-3}) are the concentration on either side of the surface. Internal boundaries representing the chloroplast envelope, mitochondria envelope and tonoplast were modelled in the same way except that the cell wall and membrane were set to be impermeable to HCO_3^-.

In the compartments of the cytosol, chloroplast, mitochondria and vacuole, reaction-diffusion processes of CO_2 were described by a general equation:

$$D_c \cdot r_{f,i} \cdot \nabla^2 C = f + h - r_d - r_p \quad \text{(S2)}$$

where D_c (m^2 s^{-1}) is the liquid-phase diffusion coefficient of CO_2 in water, $r_{f,i}$ is a dimensionless factor representing the decrease of the diffusion coefficient relative to free diffusion in water in different compartments. $\nabla^2 C$ is the Laplace operator which equals $\frac{\partial^2 C}{\partial x^2} + \frac{\partial^2 C}{\partial y^2} + \frac{\partial^2 C}{\partial z^2}$. While on the right-hand side of the equation, f is volumetric carboxylation rate (mol m^{-3} s^{-1}), h is hydration rate from CO_2 to HCO_3^- catalyzed by CA, and r_d is volumetric respiration rate, and r_p is volumetric photo-respiration rate. In addition, these terms are distributed differently in each compartment, for example, in the cytosol $f = r_d = r_p = 0$, in the chloroplast $r_d = r_p = 0$, and in mitochondria $f = 0$. The reaction-diffusion processes of HCO3- in the cytosol, chloroplast, mitochondria and vacuole were described by:

$$D_b \cdot r_{f,i} \cdot \nabla^2 B = -h \quad \text{(S3)}$$

where D_b (m^2 s^{-1}) is the liquid phase diffusion coefficient for bicarbonate, and B (mol m^{-3}) is the bicarbonate concentration.

The volumetric carboxylation rate f is calculated based on the FvCB model:

$$\begin{aligned} f &= \min(f_c, f_j) \\ &= \min\left(\frac{k_c X_c C}{C + K_m}, \frac{jC}{4C + 8\Gamma^*}\right) \end{aligned} \quad \text{(S4)}$$

Where f_c (mol m^{-3} s^{-1}) is volumetric Rubisco limited carboxylation rate, and f_j is (mol m^{-3} s^{-1}) volumetric RuBP-regeneration limited carboxylation rate. k_c (s^{-1}) is Rubisco turnover rate, and X_c (mol m^{-3}) is the Rubisco concentration. C (mol m^{-3}) is the CO_2 concentration, K_m (mol m^{-3}) is the effective Michaelis-Menten constant under specific oxygen concentrations, j (mol m^{-3}) is the volumetric electron transport rate and Γ^* (mol m^{-3}) is the compensation point of chloroplastic [CO_2] in the absence of respiration.

The volumetric photorespiration rate r_p in the mitochondria was calculated as integration over the chloroplast volume and assigned uniformly to mitochondria by dividing by the mitochondria volume (V_m) in that mesophyll cell (Eqn S5).

$$r_p = \frac{\iiint_{chl} \frac{f\Gamma^*}{C} dxdydz}{V_m} \tag{S5}$$

The volumetric respiration rate r_d, was calculated from the respiration rate per leaf area (R_d) by dividing by the mitochondria volume (Eqn S6).

$$r_d = \frac{R_d S}{\sum V_m} \tag{S6}$$

The hydration rate h (mol m^{-3} s^{-1}) was approximated by Eqn S7.

$$h = \frac{k_a X_a (C - \frac{BH}{K_{eq}})}{K_a + \frac{K_a}{K_{HCO3}} B + C} \tag{S7}$$

where k_a (s^{-1}) is the turnover rate of CA, X_a (mol m^{-3}) is the concentration of CA, H (mol m^{-3}) is the proton concentration, K_a (mol m^{-3}) and K_{HCO3} (mol m^{-3}) are the Michaelis-Menten constants of hydration and dehydration, and K_{eq} (mol m^{-3}) is the equilibrium constant.

- Output A_n and ΦPSII based on the solution from solver

A finite element method was applied to solve the reaction-diffusion system, from which the steady-state CO_2 concentration in 3D space was predicted. By integration and summation of the net photosynthesis rate in each cell, it's easy to obtain A_n for the whole leaf.

The calculation of ΦPSII in *eLeaf* is modified from the method in Evans 2009, which is used to calculate ΦPSII in a spinach model consisting of 17 paradermal layers.

The light absorbed by PSII in each cell driving linear electron transfer chain, $I^{(i)}$ is,

$$I^{(i)} = I \cdot ab^{(i)} \cdot \beta \tag{S8}$$

where I is the incident irradiance, $ab^{(i)}$ is the light absorptance of ith cell, β is the proportion of absorbed light partitioned to PSII.

Then $j_i^{(i)}$, i.e. the light limited rate of PSII electron transfer rate of ith cell, is calculated by $I^{(i)} \cdot Y(II)_{LL}$. $Y(II)_{LL}$ is the conversion efficient of photosystem II from absorbed photons into e^-.

For cells whose photosynthesis is RuBP regeneration-limited, the electron transport rate $j^{(i)}$ is calculated as

$$j^{(i)} = \mathrm{minh}(j_i^{(i)}, j_m^{(i)}) = \left\{ j_i^{(i)} + j_m^{(i)} - \left[\left(j_i^{(i)} + j_m^{(i)} \right)^2 - 4\theta j_i^{(i)} j_m^{(i)} \right]^{0.5} \right\} \Big/ 2\theta \tag{S9}$$

If the photosynthesis of ith cell is Rubisco limited, then its $j^{(i)}$ is calculated from

$$v_m^{(i)} \frac{c_c^{(i)}}{c_c^{(i)} + K_m} = j^{(i)} \frac{c_c^{(i)}}{4c_c^{(i)} + 8\Gamma^*} \tag{S10}$$

The photochemical efficiency of ith cell, $\Phi PSII^{(i)}$, is therefore

$$\Phi PSII^{(i)} = j^{(i)} / I^{(i)} \tag{S11}$$

The photochemical efficiency of the leaf, $\Phi PSII$, is measured from the fluorescence under actinic light, F_s, and the fluorescence under a saturating pulse, F_m'(Genty et al., 1989).

$$\Phi PSII = 1 - F_s \big/ F_m' \tag{S12}$$

In a similar way to Evans 2009, F_s equals to the sum of $F_s^{(i)}$ for each cell, while $F_s^{(i)}$ is approximated by

$$F_s^{(i)} = I^{(i)} \cdot c \tag{S13}$$

where c is an assumed constant.

F_m' equals the sum of $F_m'^{(i)}$ for each cell, while $F_m'^{(i)}$ is approximated by

$$F_m'^{(i)} = I^{(i)}c/(1-\Phi PSII^{(i)}) \tag{S14}$$

Eqn. S13, Eqn. S14 and Eqn. S12 combine into

$$\Phi PSII = 1 - \frac{\sum I^{(i)}c}{\sum I^{(i)}c/(1-\Phi PSII^{(i)})} \tag{S15}$$

and the assumed *c* is actually canceled out in the final expression.

In this way, with predicted $ab^{(i)}$ and $c_c^{(i)}$ from *eLeaf* together with *s*, $Y(II)_{LL}$, $v_m^{(i)}$, K_m and Γ^*, ΦPSII under different conditions can be calculated.

GENETIC ALGORITHM FOR PARAMETER ESTIMATION

A genetic algorithm (GA) is a metaheuristic search algorithm inspired by the process of natural selection. Five phases are included in a genetic algorithm, 1) initialization of population; 2) evaluate fitness; 3) selection; 4) crossover; 5) mutation, as summarized in the Workflow below.

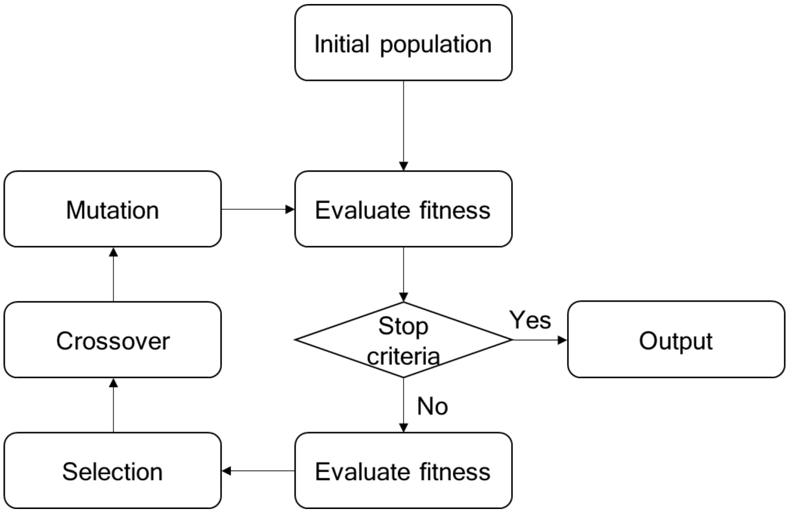

Workflow of a genetic algorithm.

- Initialization of population

eLeaf IR64-aCO_2 model and IR64-eCO_2 model are fitted simultaneously, i.e. in our case 'individual' in the genetic algorithm means a simulation of IR64-AC model and a simulation of IR64-eCO_2 model. We have 7 parameters to fit for each model (V_m, J_m, R_d, s, $Y(II)_{LL}$, [CA] and wall permeability). [CA] and wall permeability are assumed to be equal between the IR64-aCO_2 and IR64-eCO_2 models, so together we have 12 parameters to fit for each individual.

A generation of GA is one iteration of the algorithm from 'evaluate fitness' to generate a new population via 'selection', 'crossover' and 'mutation'. Here we set 12*6=72 as the population size of each generation. The recommended population size is 4-fold to 10-fold larger than the number of variables being fitted.

After setting the upper and lower boundary of each variable, an 'initial population' was generated randomly in the parameter space. Here, to increase the efficiency of searching

at the beginning, we also suggest adding an ‘individual’ obtained by fitting the physiological data under the classic FvCB model.

- Evaluate fitness

Fitness of each ‘individual’ is the reciprocal of the least squared residual of simulated A_N & ΦPSII with experimental data. Considering A_N varies between 0 to 50, while ΦPSII is always under 0.9. Direct calculation of the least squared residual will over-estimate the weight of A_N. Therefore each response curve (light and CO2 under low or normal oxygen) was normalized to its maximum value before the calculation of the least squared residual. The closer the simulation is to the experimental measurement, the higher the fitness is.

- Selection

Ten individuals are selected as parents to build the new generation of population. Normally, a genetic algorithm just take a random strategy during the selection. The probability of choosing an individual as one of the parents is proportional to its fitness. Here we made the probability of choosing one of the top 10 individuals with the largest fitness 3-fold higher, which seems accelerate the convergence of our parameter estimation.

- Crossover

A new ‘individual’ is generated from two parents first via a ‘crossover’. Imagine parent A equals [a1, a2,…, a12], parent B equals [b1, b2,…, b12], if a ‘crossover’ happens at a position between the 5th and 6th variables, there are three possible new ‘individuals’

1) [a1,…, a5, b6,…, b12]
2) [b1,…, b5, a6,…, a12]
3) [(a1+b1)/2,…, (a12+b12)/2]

These three possible new ‘individuals’ are also randomly selected during the ‘crossover’.

- Mutation

After the ‘crossover’ step, we have a new population of 12*6 individuals. Then a step of ‘mutation’ is introduced to ensure that it is theoretically possible for the GA to search the whole parameter space. A small mutation rate (5%) of each variable in each ‘individual’ is implemented. The selected “loci” of mutation will be assigned with a new value randomly from its range.

- Iteration

For our model and data, the GA converged after 50 generations, i.e. the model fitness converged. Values of parameters (V_m, J_m, R_d, s, $Y(II)_{LL}$, [CA] and wall permeability) for both models were therefore taken to further analysis.

References

Xiao, Y., Tholen, D. and Zhu, X.G. (2016) The influence of leaf anatomy on the internal light environment and photosynthetic electron transport rate: exploration with a new leaf ray tracing model. *J Exp Bot*, **67**, 6021-6035

Evans, J.R. (2009) Potential errors in electron transport rates calculated from chlorophyll fluorescence as revealed by a multilayer leaf model. Plant and Cell Physiology **50,** 698-706

USER MANUAL FOR eLEAF INSTALLATION

eLeaf installation

Currently *eLeaf* (v1.2.5) only supports the Linux operation system. A module of automatic 3D reconstruction is implemented by a software called COMSOL Multiphysics and coded in MATLAB scripts. The connection between COMSOL Multiphysics and MATLAB is provided by a package in COMSOL Multiphysics called LiveLink for MATLAB. The module of ray tracing is coded in C based on the triangle meshed surface of reconstructed 3D geometry. The CO_2 reaction-diffusion module is implemented again in COMSOL Multiphysics and coded in MATLAB scripts. All the modules are automatically driven by a master script in MATLAB (**run_e_leaf_v1_2_5.m**).

Preparation:

- a Linux system (we used CentOS 7)

- install gcc library for the ray tracing in the light propagation module (we used version 4.8.5-28)

- install MATLAB (we used version 2016)

- install COMSOL Multiphysics (we used version 5.3, with packages of CFD, LiveLink for MATLAB and LiveLink for CAD)

Run *eLeaf*

- Start COMSOL Multiphysics in the server mode

- Start MATLAB and connect to COMSOL server

- In MATLAB, switch to directory eleaf/1.e_geom/.

- To repeat the eLeaf IR64-aCO_2 model, type
    - run_e_leaf_v1_2_5(4,[0 0 0 0 0 0 0 0 0])
- To repeat the eLeaf IR64-eCO_2 model, type
    - run_e_leaf_v1_2_5(4,[1 1 1 1 1 1 1 1 1])

- The first input number “4” here configures an operating mode, meaning *eLeaf* will run the simulation under the same light and C_i conditions as the experimental data. The second input is a vector configuring values assigned to each category of parameters (8 categories in all). 0 means the model will use values from the IR64-AC model, 1 means the model will use values from the IR64-EC model.
- To dissect the contributions of anatomical and biochemical features, new F_{1-9} models are created based on combinations of the IR64-AC model and the IR64-EC model. To simulate, for example, the F_1 model, type

```
run_e_leaf_v1_2_5(2,[1 0 0 0 0 0 0 0 0])
```

The input number “2” here configures the operating mode of *eLeaf* for parameter dissection under the same light and CO_2 conditions.

Inputs and Outputs of *eLeaf*

Inputs in Table 1 and Table 2 are assigned in **e_geo_parainput_v1_2_5_a4tfit.m**

Experiment data are in **2.5.eleaf_fvcb_fit/export_meas4comsol_new_selected.m.** We measured the CO_2 response and light response under ambient oxygen, correspondingly there are two studies for *eLeaf* simulation:

- Study 1 is the light response curve.
- Study 2 is the CO_2 response curve.

Outputs will be in folder **eleaf/2.5.eleaf_fvcb_fit/**, including

- a mph file (COMSOL model file) named **“eleaf_fvcb_prswp_CKIR64_a_phipsii.mph”** or **“eleaf_fvcb_prswp_HCIR64_a_phipsii.mph”**, which can be opened in COMSOL GUI. This is a file of the model with steady state solutions of both studies.
- **“results_merged_475nm_*”**, **“results_merged_625nm_*”** and **“ab_profile.mat”** are light absorption profiles under the wavelength of 475nm and 625nm predicted from the ray tracing module.
- **“a_std1_prswp_CKIR64.txt”** and **“a_std2_prswp_CKIR64.txt”** are exported files from solutions in the mph file. Predicted leaf photosynthesis rate under different conditions are extracted and recorded.

- **“phipsii_std1_prswp_CKIR64.txt”** and **“phipsii_std2_prswp_CKIR64.txt”** are exported files from solutions in the mph file. Predicted carboxylation rate and Cc of each cell under different conditions are extracted and recorded. ΦPSII of the leaf can be calculated based on these files.

Developer guide for *eLeaf* code

Automatic 3D reconstruction module

The current version of *eLeaf* (v1.2.5) has only been tested with two sets of parameters from IR64-aCO_2 and IR64-eCO_2 plants. Therefore the author can only ensure that within these ranges, the *eLeaf* model can run successfully. Extreme input values such as a very small intercellular air space, or a very big or small plastid volume may lead to warnings in the MATLAB command window or even crash the model.

Light propagation module

Information of the boundaries for the ray tracing simulation needs to be passed from the module of 3D reconstruction. For each new 3D geometry, a new **2.e_raytracing/Defs.h** file will be generated correspondingly. The triangle meshed surfaces of the reconstructed 3D geometry are represented in the ply format, which are generated by the function **“2.e_raytracing/geo_export/geo_export_e_geo_main_v1_2.m”**. Commands compiling and running the ray tracing are included in file **run_e_leaf_v1_2_5.m**. Light properties such as wavelength and absorption coefficients are configured there. Refractive indexes of different materials are set in **“Defs_template.h”** (line 119-123). Light absorption profiles are simulated under red light (625 nm) and blue light (475 nm), because the LED light source in the infra-red gas exchange analysis system is composed of 90% of red light and 10% of blue light. Merging of the different light absorption profiles under different wavelengths is achieved by **”2.5.eleaf_fvcb_fit/e_pre_physics_v1_2_3.m”.**

CO_2 reaction-diffusion module

Biophysical and biochemical properties of each domain and each boundary in the 3D geometry are automatically assigned in *eLeaf*. Most of these parameters are assigned in **2.5.eleaf_fvcb_fit/e_physics_v1_2_3_fvcb.m**, only the scale factor for the permeability of cell wall and concentration of CA are in the master input file **e_geo_parainput_v1_2_5_a4tfit.m**. Solver configurations, extraction and export of solutions to output files are in **2.5.eleaf_fvcb_fit/e_study_v1_2_3_prswp_forfvcb_fit.m.**

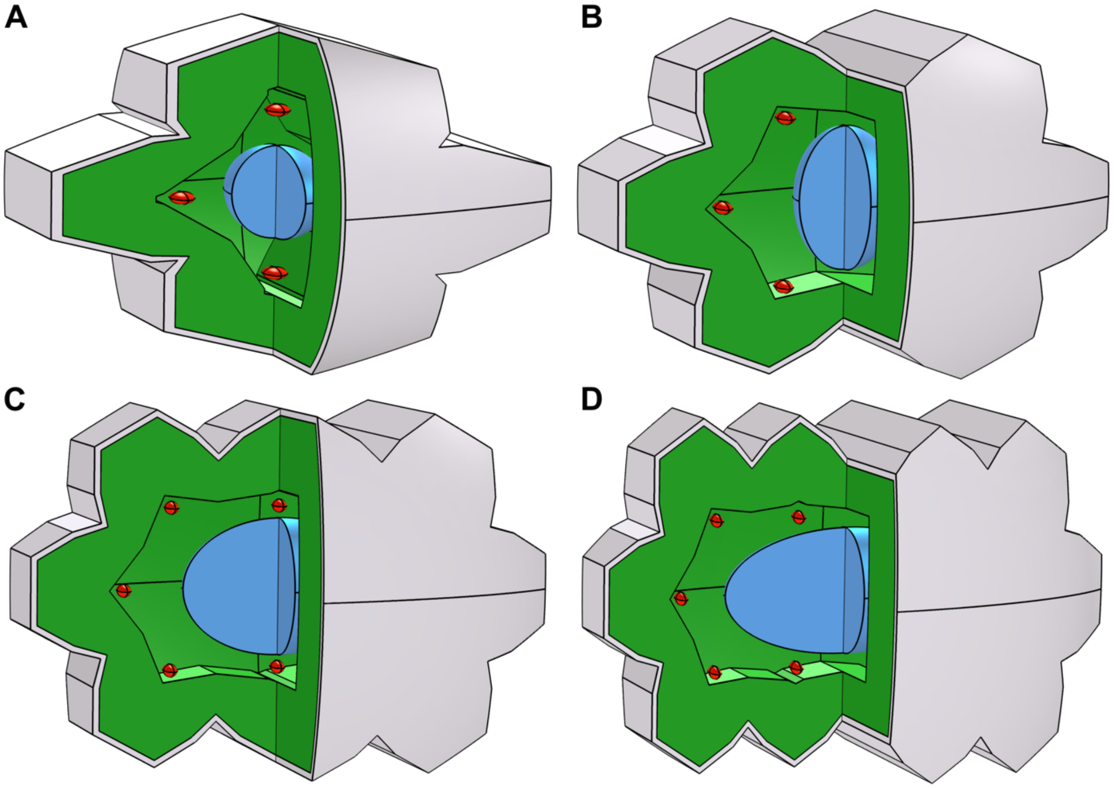

Supplementary Figure S1. Varying lobe number in the eLeaf model
(**A-D**) Mesophyll cells with 4, 6, 8 and 10 lobes were used to implement the 3D leaf models corresponding to outputs shown in Figure 6E,F. Other values in the model, including cell length, width and volume, as well as %age plastid content, were maintained to the same value in all lobe variants as far as possible within model constraints.

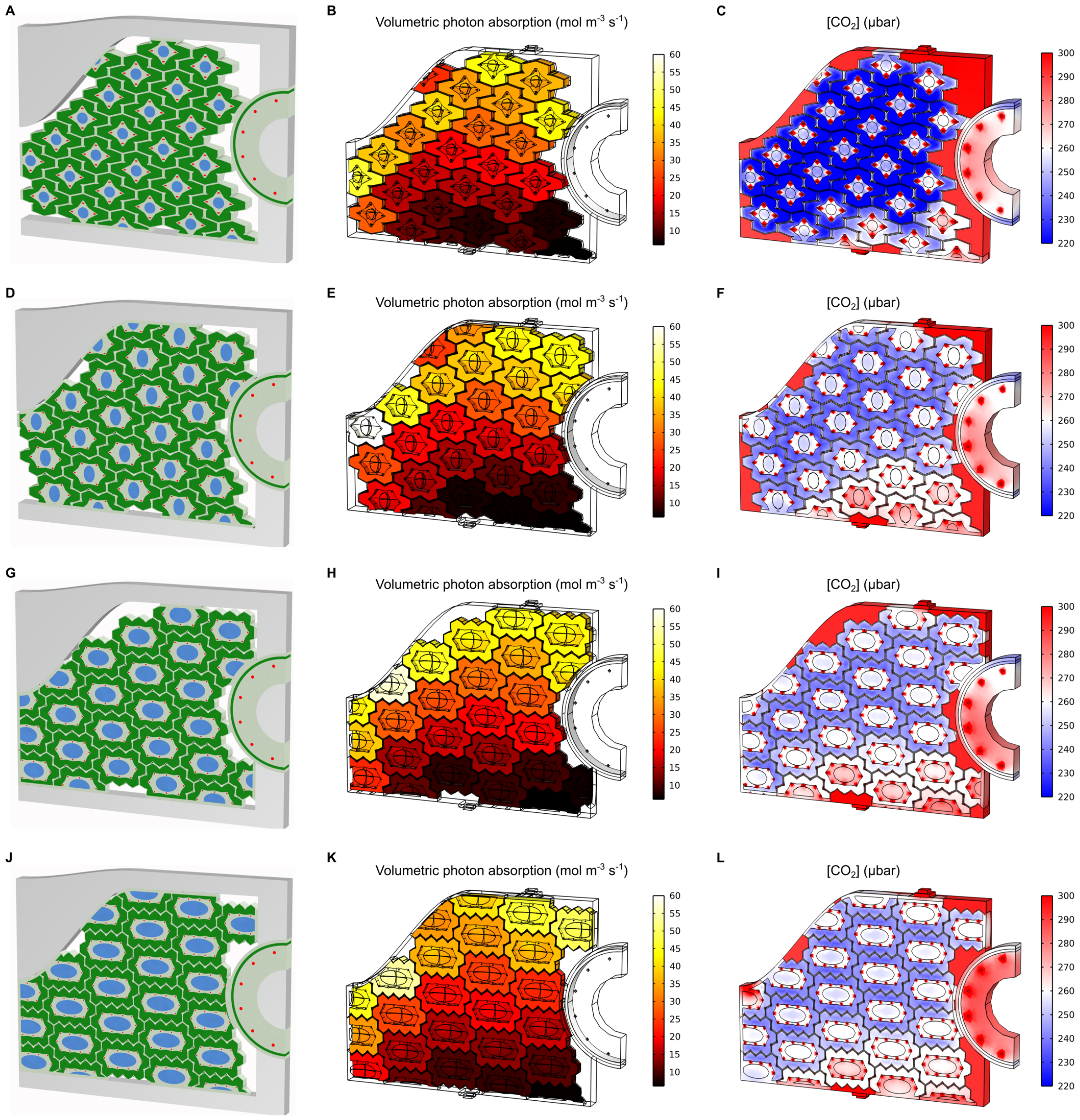

Supplementary Figure S2. Sensitivity analysis to mesophyll cell lobing (**A,D,G,J**) eLeaf models filled with mesophyll cells with 4, 6, 8 and 10 lobes, respectively. (**B,E,H,K**) Predicted light absorption by chloroplasts. The incident irradiance is 1000 µmol m^{-2} s^{-1}. Color bar represents the volumetric photon absorption (mol photons m^{-3} s^{-1}) within each cell. (**C,F,I,L**) CO_2 concentration within each cell simulated with C_i = 300 µbar and irradiance= 1000 µmol m^{-2} s^{-1}. For each scenario, 10 model replicates were simulated and the mean value shown. Colour bar represents the CO_2 level (µbar).

Supplementary Table 1| Acronyms, definition, variables, and units used

Symbol	Definition	Units
A_N	Net photosynthesis rate of a leaf	μmol m^{-2} s^{-1}
C_i	Intercellular CO_2 partial pressure	μbar
V_{cmax}	CO_2-saturated carboxylation rate of a leaf	μmol m^{-2} s^{-1}
V_{omax}	O_2-saturated oxygenation rate of a leaf	μmol m^{-2} s^{-1}
J	Photosynthetic electron transfer rate under saturated $NADP^+$ and ADP	μmol m^{-2} s^{-1}
J_m	Light saturated electron transfer rate under the CO_2 concentration of light curves	μmol m^{-2} s^{-1}
Y(II)	Yield of photosystem II; also known as *ΦPSII*	Dimensionless
$Y(II)_{LL}$	Initial Y(II) extrapolated at PPFD = 0 μmol m^{-2} s^{-1}	Dimensionless
Ab.	Light absorptance of a leaf; $ab^{(i)}$ represents light absorptance of ith chloroplasts	Dimensionless
β	Proportion of absorbed PPFD partitioned to photosystem II. $J = PPFD \times Ab. \times \beta \times Y(II)$	Dimensionless
s	$Ab. \times \beta$, which lumps leaf light absorption and partition by photosystem II	Dimensionless
θ	Curvature of the non-rectangular hyperbola describing the PPFD dependence of *J* for each chloroplast	Dimensionless
R_d	Respiration rate of a leaf in the light	μmol m^{-2} s^{-1}
$S_{c/o}$	Rubisco specificity factor; $S_{c/o} = (V_{cmax} \times K_o)/(V_{omax} \times K_c)$	Dimensionless
K_c	Rubisco Michaelis-Menton constant for CO_2	μbar
K_o	Rubisco Michaelis-Menton constant for O_2	μbar
$[CA]_c$	CA concentration in cytosol; default value $0.5 \times [CA]_s$	mol m^{-3}
$[CA]_s$	CA concentration in stroma; default value 0.27	mol m^{-3}
G_{wall}	Wall conductance for CO_2. default value 0.09 mol m^{-2} s^{-1} bar^{-1} for 1.5 μm cell wall	μmol m^{-2} s^{-1}

Supplementary Table 2: Structural parameters for the *eLeaf* model. Measurements of rice leaves were made under either ambient CO_2 (aCO2) or elevated CO_2 (eCO2) and classified into 7 categories (F). Values are means (with standard deviation in brackets). For all data categories, n =5 except for category F3 and F7, n = 15. Relative difference was calculated as (eCO_2-aCO_2)/aCO_2 (%)). BS= bundle sheath; MC = mesophyll cell. S_{mes} = exposed mesophyll cell area (%).

**Parameter**	**F**	**aCO2** (s.d)	**eCO2** (s.d)	**Relative difference** (%)
Leaf thickness at minor vein (μm)	1	75.52 (8.17)	77.99 (7.60)	3.27
Leaf thickness at bulliform cells (μm)	1	70.5 (10.91)	71.30 (4.70)	1.14
Mesophyll thickness at minor vein (μm)	1	64.42 (6.81)	67.41 (7.69)	4.64
Mesophyll thickness at bulliform cells (μm)	1	31.4 (5.84)	27.91 (4.39)	-11.12
Distance between two minor veins (μm)	1	186.5 (15.4)	200.4 (18.9)	7.45
BS layer thickness (μm)	2	9.65 (0.97)	9.01 (0.34)	-6.64
BS layer area (μm^2)	2	870.2 (134.5)	657.6 (58.2)	-24.4
Plastid area in BSCs (%)	2	10.44 (1.18)	11.76 (1.09)	12.6
Mesophyll porosity (%)	3	8.02 (1.89)	6.17 (1.10)	-23.07
MC length (μm)	4	18.98 (1.57)	19.78 (1.15)	4.20
MC width (μm)	4	13.25 (0.70)	13.35 (1.00)	0.75
MC volume (μm^3)	4	1887 (244)	1909 (213)	1.16
Plastid area in MCs (%)	5	69.71 (3.57)	65.34 (5.05)	-6.27
Mesophyll cell wall thickness (μm)	6	0.218 (0.030)	0.230 (0.02)	5.70
S_{mes} (%)	7	68.37 (4.60)	57.99 (4.91)	-15.19

Supplementary Table 3: Metabolic and physiology parameters for the *eLeaf* model. Measurements of rice leaves were made under either ambient CO_2 (aCO2) or elevated CO_2 (eCO2) or are values from the literature. Relative difference was calculated as (eCO_2-aCO_2)/aCO_2 (%)). F refers to the category number in the *eLeaf* model. Definitions of the parameters are given in Supplemental Table 1. For chlorophyll analysis, n =8, with mean value shown.

	**Parameter**	**F**	**Unit**	**aCO2**	**eCO2**	**Relative change** (%)
Measured	Chlorophyll	8	mg mm^{-2}	5.43e-4	5.51e-4	1.47
FvCB-type metabolic model	V_m	9	µmol m^2 s^{-1}	114.39	121.98	6.64
	J_m	9	µmol m^{-2} s^{-1}	224.63	268.79	19.66
	$Y(II)_{LL}$	9	mole e^- per mole of photon	0.63	0.61	-3.17
	s	9	Dimensionless	0.63	0.48	-23.81
	R_d	9	µmol m^{-2} s^{-1}	2.92	3.34	14.38
	$S_{c/o}$	Const.	µbar	3375	3375	/
	K_c	Const.	µbar	239	239	/
	K_o	Const.	µbar	26600	26600	/
	ϑ	Const.	Dimensionless	0.98	0.98	/
Other factors fitted	Scale factor for [CA] in cytosol and stroma	Const.	Dimensionless	1.26	1.26	/
	Scale factor for cell wall permeability	Const.	Dimensionless	2.27	2.27	/

Supporting Information Video: Generation of the *eLeaf* model

(see separate file)

The eLeaf model consists of a series of virtual cells bounded by parameters of leaf and mesophyll thickness and interveinal distance set by data obtained from hand-sections of rice. Bundle sheath size and plastid volume, as well as vein size obtained from TEM images are used to set these parameters, with mesophyll cell size and lobing data obtained from confocal light microscopy. Mesophyll cell wall thickness, plastid volume and exposed mesophyll cell area are obtained from TEM images, with mesophyll cell shape and separation adjusted to match overall mesophyll porosity calculated by microCT imaging of rice leaves
